# miRNA‐106a and prostate cancer radioresistance: a novel role for LITAF in ATM regulation

**DOI:** 10.1002/1878-0261.12328

**Published:** 2018-06-14

**Authors:** Christianne Hoey, Jessica Ray, Jouhyun Jeon, Xiaoyong Huang, Samira Taeb, Jarkko Ylanko, David W. Andrews, Paul C. Boutros, Stanley K. Liu

**Affiliations:** ^1^ Biological Sciences Sunnybrook Research Institute Sunnybrook Health Sciences Centre Toronto Canada; ^2^ Department of Medical Biophysics University of Toronto Canada; ^3^ Ontario Institute for Cancer Research University Health Network Toronto Canada; ^4^ Department of Pharmacology and Toxicology University of Toronto Canada; ^5^ Department of Radiation Oncology University of Toronto Canada

**Keywords:** ATM, KU‐55933, LITAF, miR‐106a, prostate cancer, radiation resistance

## Abstract

Recurrence of high‐grade prostate cancer after radiotherapy is a significant clinical problem, resulting in increased morbidity and reduced patient survival. The molecular mechanisms of radiation resistance are being elucidated through the study of microRNA (miR) that negatively regulate gene expression. We performed bioinformatics analyses of The Cancer Genome Atlas (TCGA) dataset to evaluate the association between miR‐106a and its putative target lipopolysaccharide‐induced TNF‐α factor (LITAF) in prostate cancer. We characterized the function of miR‐106a through *in vitro* and *in vivo* experiments and employed transcriptomic analysis, western blotting, and 3′UTR luciferase assays to establish LITAF as a *bona fide* target of miR‐106a. Using our well‐characterized radiation‐resistant cell lines, we identified that miR‐106a was overexpressed in radiation‐resistant cells compared to parental cells. In the TCGA, miR‐106a was significantly elevated in high‐grade human prostate tumors relative to intermediate‐ and low‐grade specimens. An inverse correlation was seen with its target, LITAF. Furthermore, high miR‐106a and low LITAF expression predict for biochemical recurrence at 5 years after radical prostatectomy. miR‐106a overexpression conferred radioresistance by increasing proliferation and reducing senescence, and this was phenocopied by knockdown of LITAF. For the first time, we describe a role for miRNA in upregulating ATM expression. LITAF, not previously attributed to radiation response, mediates this interaction. This route of cancer radioresistance can be overcome using the specific ATM kinase inhibitor, KU‐55933. Our research provides the first report of miR‐106a and LITAF in prostate cancer radiation resistance and high‐grade disease, and presents a viable therapeutic strategy that may ultimately improve patient outcomes.

**Abbreviations**
A‐T: ataxia telangiectasia
ATM: ataxia telangiectasia mutated
BCR: biochemical recurrence
DDR: DNA damage repair
DSB: double‐stranded break
IRR: radiation‐resistant
LITAF: lipopolysaccharide‐induced TNF‐α factor
NGS: next‐generation sequencing
RBL‐2: retinoblastoma‐like protein 2
SA: senescence‐associated
SBRT: stereotactic body radiotherapy
TCGA: The Cancer Genome Atlas
UTR: untranslated region

## 1 Introduction

Prostate cancer is the most common nonmelanoma skin cancer affecting men, and a leading cause of cancer‐related death worldwide. Radiation is a primary treatment modality for localized prostate cancer, with equivalent survival outcomes for low‐ and intermediate‐risk patients compared to radical prostatectomy (Hamdy *et al*., 2016). However, despite administration of escalated doses of radiation to the prostate, high‐risk patients with prostate cancer are at a significantly higher risk of disease relapse (Chang *et al*., 2014). Therefore, there is an unmet need to better characterize and treat tumors at an elevated risk of relapse after radiotherapy.

MicroRNA (miR) are small (20–25 nucleotide) noncoding RNA that negatively regulate gene expression. miRNA are transcribed into long stem loop structures that ultimately form a double‐stranded RNA. The dominant RNA strand is incorporated into the RNA silencing complex to facilitate target mRNA binding, and the complementary strand is degraded (He and Hannon, 2004). miRNA decrease protein expression of their target mRNA by binding imperfectly to 6–7 nucleotides in the seed sequence in the mRNA 3′ untranslated region (UTR) (He and Hannon, 2004). Transcript degradation is the major outcome of miRNA‐mRNA binding, occurring through the 5′‐to‐3′ mRNA decay pathway (Huntzinger and Izaurralde, 2011).

miRNA are involved in all cell processes and have been of particular interest due to their role in cancer. They have been implicated in cell cycle progression, metastatic progression, and treatment response (Huang *et al*., 2013, 2015; Mesci *et al*., 2017). Individual miRNA can regulate thousands of cellular pathways simultaneously (Huntzinger and Izaurralde, 2011). Small perturbations in miRNA expression could lead to harmful downstream effects resulting in tumorigenesis and treatment resistance. This multimodal action of miRNA on cellular processing makes them a particularly promising target for cancer treatment.

We identified miR‐106a (dominant strand is miR‐106a‐5p, henceforth referred to as miR‐106a) from a next‐generation sequencing (NGS) screen of radiation‐resistant (IRR) PC3 cells, as previously described by our group (Huang *et al*., 2013). Bioinformatics analyses of The Cancer Genome Atlas (TCGA) dataset revealed that miR‐106a was significantly higher in prostate tumors compared to normal prostate. miR‐106a showed significantly higher expression in high‐grade prostate tumors compared to low‐ and intermediate‐grade tumors, suggesting that miR‐106a may be involved in disease progression. We discovered that miR‐106a increased survival and growth after ionizing radiation using *in vitro* and *in vivo* prostate cancer models. We also confirmed that miR‐106a targets lipopolysaccharide‐induced TNF‐α factor (LITAF), to produce this radioresistant phenotype. In addition, we found that high miR‐106a and low LITAF expression predict for decreased BCR‐free survival at 5 years after radical prostatectomy. In this study, we describe for the first time two novel players in prostate cancer radioresistance: miR‐106a and LITAF. We describe a novel mechanism where miR‐106a overexpression and LITAF knockdown upregulate ataxia telangiectasia‐mutated (ATM) expression. Inhibition of ATM kinase activity with KU‐55933 resensitized miR‐106a‐overexpressing cells to radiation, highlighting a promising therapeutic intervention for miR‐106a radioresistant prostate cancer.

## 2 Materials and methods

### 2.1 Bioinformatics analysis

Expression data and clinical information from 487 prostate tumor samples and 52 matched normal samples were downloaded from the TCGA (https://portal.gdc.cancer.gov). Of these, 287 samples were Gleason score ≤7 (low‐ and intermediate‐grade) and 200 samples were Gleason >7 (high‐grade) disease. Analyses were carried out in the programming language r (v3.4.0, Auckland, New Zealand). Two‐sided Wilcoxon tests were used to examine statistical significance for group comparisons. Data visualization employed the bpg package (v5.7.1, Toronto, ON, Canada) (P'ng *et al*. in submission, https://www.biorxiv.org/content/early/2017/06/26/156067).

For biochemical recurrence (BCR)‐free survival analysis, 391 TCGA tumor samples showing BCR within 5 years were used. Expression values of miR‐106a and LITAF were dichotomized (high *vs*. low) with the cutoff values showing the most discriminative power in the univariate cox model for BCR‐free survival. survmisc r package (version 0.4.5, Pheonix, AZ, USA) was used to optimized cutoff values. Kaplan‐Meier method was used to estimate survival distributions, and the relationship between BCR‐free survival and each variable (miR‐106a and LITAF) was analyzed with Wald test. The relationship between BCR‐free survival and four subgroups (combinations of expressions of miR‐106a and LITAF) was analyzed with log‐rank test.

### 2.2 Cell culture

PC3 and DU145 prostate adenocarcinoma cell lines were purchased from the American Type Culture Collection (ATCC, USA). Cell lines were maintained as previously described by our group (Huang *et al*., 2013) and tested regularly for mycoplasma contamination using the MycoAlert™ Mycoplasma Detection Kit (Lonza, Mississauga, ON, Canada).

### 2.3 Transfection of miR‐106a mimic and LITAF siRNA

A total of 2 × 10^5^ cells were seeded into 6‐well plates. Twenty‐four hours later, miRNA mimics (5 μm) or 3‐sequence pooled siRNA (10 μm) (Shanghai GenePharma Co., Shanghai, China) were transfected into cells using Lipofectamine 2000 Reagent (Invitrogen, Thermo Fisher Scientific, Mississauga, ON, Canada) and Opti‐MEM I (1X) reduced serum media (Invitrogen), as per manufacturer's recommendations. Cells were collected at 24 h post‐transfection for all experiments. miRNA mimic and siRNA sequences can be found in Table S1A.

### 2.4 Radiation

Radiation was performed using a Faxitron 43855F X‐ray Irradiator (Faxitron Bioptics LLC, Tucson, AZ, USA). Radiation settings were as follows: 160 kV, 6.3 mA, for 1.1, 2.3, 3.5, 4.6, and 5.7 min for a radiation dose of 2, 4, 6, 8, and 10 Gy, respectively.

### 2.5 RNA isolation

Cells were lysed. Total RNA enriched in miRNA and mRNA was collected using miRVANA microRNA isolation kit (Life Technologies, Thermo Fisher Scientific) and RNeasy Mini Kit (Qiagen, Toronto, ON, Canada), respectively. RNA concentration and purity were determined using the Agilent 2100 Bioanalyzer with the RNA 6000 Nano kit (Agilent Technologies Canada Inc., Mississauga, ON, Canada).

### 2.6 qRT‐PCR cDNA synthesis

miRNA was synthesized into cDNA using miSCRIPT II RT Kit (Qiagen); mRNA was made into cDNA using the SuperScript^®^ VILO™ kit (Invitrogen). Real‐time quantitative PCR was performed to analyze gene expression using the miScript^®^ SYBR^®^ Green PCR Kit for miRNA transcripts (Qiagen), and SYBR^®^ Select Master Mix for mRNA transcripts (Life Technologies). Primer sequences are available in Table S1B (Invitrogen). Gene expression was calculated based on the comparative Ct method by Applied Biosystems StepOnePlus Real‐Time PCR System (Thermo Fisher Scientific), and relative miRNA and mRNA expression levels normalized to SNORD61_11 (SNORD) and GAPDH, respectively.

### 2.7 Clonogenic survival assay

Mimic or siRNA‐transfected PC3 and DU145 cells were seeded in triplicate, irradiated, and stained as previously described (Huang *et al*., 2013). Number of colonies (>50 cells) was counted, and surviving fraction was determined from plating efficiency of radiation‐treated cells relative to mock‐irradiated cells. Clonogenic survival was represented in dose–response curves by fitting relative surviving fraction to the linear quadratic formula equation *S = e* ^*−*α*D−*β*D2*^ using graphpad prism 5.0 (GraphPad Software, San Diego, CA, USA) (Huang *et al*., 2013). This experiment was performed three separate times.

### 2.8 Proliferation assay

Mimic or siRNA‐transfected cells were seeded in triplicate in 6‐well plates (0 Gy at 5 × 10^4^ cells per well and 8 Gy at 1 × 10^5^ cells per well) and irradiated 24 hr later. Cells were trypsinized four days later (0 Gy) or five days later (8 Gy), and viable cells were measured with the Countess automated cell counter (Invitrogen). The number of viable cells was averaged among three biological replicates.

### 2.9 Cell cycle distribution

Cells were seeded at a density of 2 × 10^5^ cells per 6‐well plate. Cells were transfected the following day with control/miR‐106a mimic and irradiated 24 hr after transfection (PC3 at 6 Gy, DU145 at 10 Gy). Cells were collected at 24 h and fixed as previously described (Huang *et al*., 2013). FACS Calibur flow cytometer (BD Biosciences, Mississauga, ON, Canada) was used to capture 10 000 events per sample, and cell cycle distribution was analyzed using FlowJo 10.0.4 (FlowJo LLC, Ashland, OR, USA). This experiment was performed three separate times.

### 2.10 Cell senescence assay

Transfected cells were seeded in a 6‐well plate (0 Gy at 1 × 10^5^ cells per well and 6 Gy at 5 × 10^4^ cells per well) and irradiated 24 hr later. Cells were fixed and stained (on day of mock irradiation for 0 Gy and 7 days after 6 Gy irradiation) for senescence‐associated (SA)‐β‐galactosidase using the Senescence β‐Galactosidase Staining Kit (Cell Signaling Technology, Whitby, ON, Canada) as per manufacturer's instructions. A minimum of 250 cells (from at least four nonoverlapping images) were counted per condition. The percentage of SA‐β‐galactosidase‐positive cells was averaged among three biological replicates.

### 2.11 Western blotting

Cells were lysed with NP‐40 lysis buffer, and total cell proteins were collected as previously described (Huang *et al*., 2013). Protein lysates (40 μg) were run on polyacrylamide gel (Bio‐Rad, Mississauga, ON, Canada) and transferred onto polyvinylidene fluoride membranes (Thermo Scientific). Membranes were blocked with 5% nonfat dry milk or 5% bovine serum albumin (Roche Diagnostics, Mississauga, ON, Canada) in TBS Tween‐20 (TBS‐T) as previously described (Huang *et al*., 2013). After overnight primary antibody incubation, membranes were washed with TBS‐T and incubated with horseradish peroxidase‐conjugated secondary antibody. Blots were visualized using ECL substrate (Bio‐Rad). All antibodies were purchased from Cell Signaling Technology, with working concentrations used as per manufacturer's instructions. Each western blotting experiment was performed in three biological replicates.

### 2.12 KU‐55933

KU‐55933 (Cat#: SML1109; Sigma‐Aldrich, Oakville, ON, Canada) was dissolved in DMSO, and cells were treated with vehicle (DMSO) or KU‐55933.

### 2.13 Target detection

Total RNA was collected from DU145 control and DU145 miR‐106a‐transfected cells using RNeasy Mini Kit (Qiagen). Gene expression analysis was performed by The Centre for Applied Genomics (The Hospital for Sick Children, Toronto, Canada) using an Affymetrix^®^ GeneChip Human Gene 2.0 ST array (Affymetrix, Thermo Fisher Scientific). Data were normalized using the default parameters in Affymetrix^®^ Expression Console™ Software 1.4. Genes downregulated to 0.8‐fold in miR‐106a compared to control were identified as possible targets. Tumor suppressor genes were isolated and cross‐referenced to miR‐106a targets yielded from a combination of *in silico* prediction algorithms: miRwalk, miRDB, PITA, MicroT4, miRMap, RNA22, miRanda, miRNAMap, RNAhybrid, miRBridge, PICTAR2, TargetScan. Resulting mRNA were checked for miR‐106a binding using TargetScan *in silico* algorithm prediction software (Agarwal *et al*., 2015), yielding 12 possible gene targets of miR‐106a. Gene expression of potential targets in cells transfected with control or miR‐106a mimic was assessed by qRT‐PCR.

### 2.14 LITAF 3′UTR luciferase reporter assay

PC3 cells were transiently co‐transfected with wild‐type or mutant (predicted miR‐106a‐LITAF seed sequence binding site nucleotides were mutated) LITAF 3′UTR luciferase reporter plasmid, β‐galactosidase‐expressing PCCALL2/anton vector (gift from J. Filmus laboratory), and control or miR‐106a mimics. Cell lysates were harvested 24 h later. Luciferase Assay Reagent (Promega, Madison, WI, USA) was added, and fluorescence was measured with a luminometer to quantify firefly luciferase activity. β‐galactosidase activity was measured by absorbance at 405 nm using the FilterMax F5 Microplate Reader. Firefly luciferase activity was normalized to β‐galactosidase activity for transfection efficiency control. This experiment was performed three separate times.

### 2.15 Stable cell line generation

Pre‐miR‐106a was cloned into a pBabe‐puro vector. miR‐106a‐pBabe and empty‐pBabe vector were transfected into Phoenix‐AMPHO retroviral packaging cell line (ATCC) and incubated for 24 h. The same day, 2 × 10^5^ DU145 cells were seeded into a 6‐well plate. The following day, 2 mL of viral media, 5 μg polybrene (Sigma‐Aldrich), and 0.5 mL FBS were added onto cells. Cells were spin transduced at 1000 g for 90 min at 37 °C. Three days later, transduced cells were selected using puromycin (1 μg·mL^−1^) for 2 weeks to generate DU145‐control or DU145‐miR‐106a stable cell lines. miR‐106a overexpression and radioresistant phenotype were confirmed by qRT‐PCR and clonogenic survival assays, respectively.

### 2.16 Tumor xenografts and radiation response *in vivo*

Six‐ to seven‐week‐old female athymic nude mice were injected subcutaneously into the right flank with three million DU145‐control or DU145‐miR‐106a stable cells and Matrigel. Tumors were measured three times a week with calipers. Tumor volume was calculated assuming an ellipsoid shape, using the formula: volume (mm^3^) = (length × width^2^)/2. When tumor volumes reached an average of 100 mm^3^ per group, mice were randomly assigned to mock irradiation or one‐time 6 Gy irradiation treatment, which is within the range of ionizing radiation used for stereotactic body radiotherapy (SBRT) treatment of prostate tumors. Our endpoint was defined as tumor volume reaching 300 mm^3^. All animal experimental procedures were performed in accordance with the University of Toronto and Sunnybrook Research Institute guidelines using a peer‐reviewed Animal Use Protocol. The Sunnybrook Research Institute Animal Care Committee (SRI ACC) has approved the Animal Use Protocol (AUP) for this study (AUP#: 14‐509).

### 2.17 Immunohistochemistry

Tumor xenografts were sectioned into 5‐μm‐thick sections. Sections were stained for hematoxylin and eosin (H&E) (Leica Biosystems, Concord, ON, Canada). Necrotic areas were outlined on tumor sections and quantitated as a percentage of total section area using the imagej software (*n* = 3 tumors per group). Proliferation was (Bethesda, MD, USA) assessed by Ki‐67 immunostaining and quantitated using imagej.

### 2.18 ATM promoter luciferase reporter assay

PC3 and DU145 cells were transiently co‐transfected with ATM promoter or control vector, β‐galactosidase PCCALL2/anton vector, and control/miR‐106a mimic or control/LITAF siRNA. Cell lysates were collected 24 hr after transfection, and luciferase activity was assessed using the LightSwitch Luciferase Assay System (SwitchGear Genomics, Active Motif, USA). ATM/control promoter luciferase constructs were used with LightSwitch™ Luciferase Assay Reagent (SwitchGear Genomics). This was performed in three biological replicates.

### 2.19 Statistical analysis

Statistical analysis was performed using the graphpad prism v5.01 software (GraphPad Software). Unpaired, two‐sided Student's *t‐*tests were used to compare the mean values between two groups. Data are represented as mean values ± SEM unless otherwise mentioned. Statistical significance was defined as *P *<* *0.05.

## 3 Results

### 3.1 miR‐106a is overexpressed in human prostate tumors and associated with biochemical recurrence

**Figure 1:**
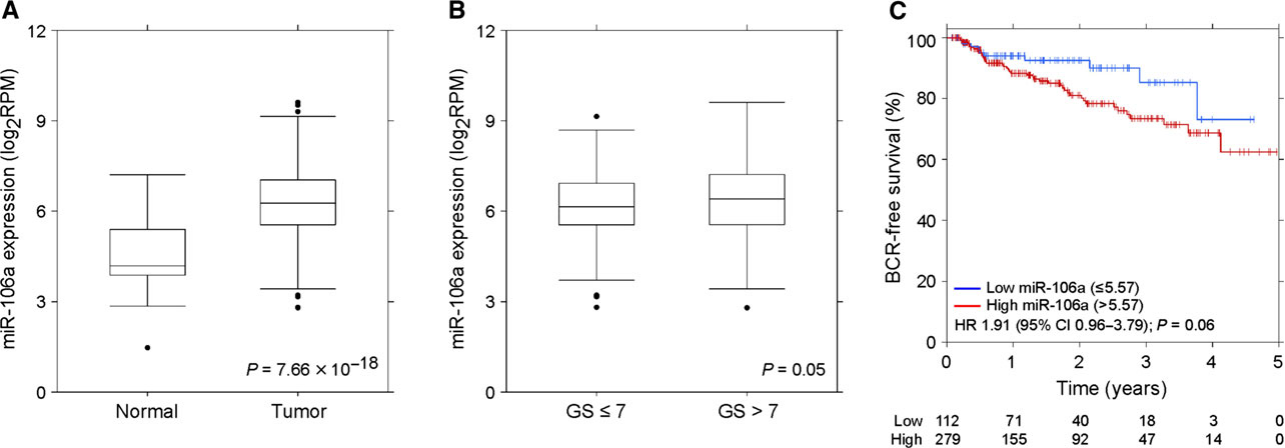
miR‐106a is aberrantly expressed in human prostate tumors. (A) Expression of miR‐106a is significantly increased in prostate tumor samples compared to normal prostate tissue (*P = *7.66 × 10^−18^; *n* = 487 tumor and *n* = 52 normal matched; two‐sided Wilcoxon test). (B) miR‐106a expression is significantly higher in tumors with Gleason score (GS) >7 compared to GS ≤7 (*P *=* *0.05; *n* = 287 for GS ≤7 and *n* = 200 for GS >7; two‐sided Wilcoxon test). (C) High miR‐106a expression is associated with lower BCR‐free survival at 5 years (391 tumors; *P *=* *0.06; Wald test).

We analyzed the TCGA dataset to determine whether miR‐106a was more abundant in prostate cancer (Cancer Genome Atlas Research Network, 2015). We found that miR‐106a was significantly overexpressed in prostate cancer (487 samples) compared to matched normal samples (52 samples; *P *= 7.66 × 10^−18^; two‐sided Wilcoxon test; Fig. 1A). miR‐106a showed significantly higher expression in clinical high‐grade (200 samples; Gleason >7) compared to low‐ and intermediate‐grade (287 samples; Gleason ≤7) tumors (*P *=* *0.05; two‐sided Wilcoxon test; Fig. 1B). This suggests that miR‐106a is involved in prostate tumorigenesis and tumor progression.

We further analyzed the TCGA dataset to determine whether miR‐106a expression correlates with biochemical recurrence (BCR)‐free survival at 5 years. Using the Kaplan‐Meier method, we determined that high miR‐106a expression is associated with lower BCR‐free survival at 5 years after radical prostatectomy (391 tumors; *P *=* *0.06; Wald test; Fig. 1C). This suggests that high miR‐106a expression is associated with poor patient outcomes.

### 3.2 miR‐106a confers an aggressive and radioresistant phenotype

**Figure 2:**
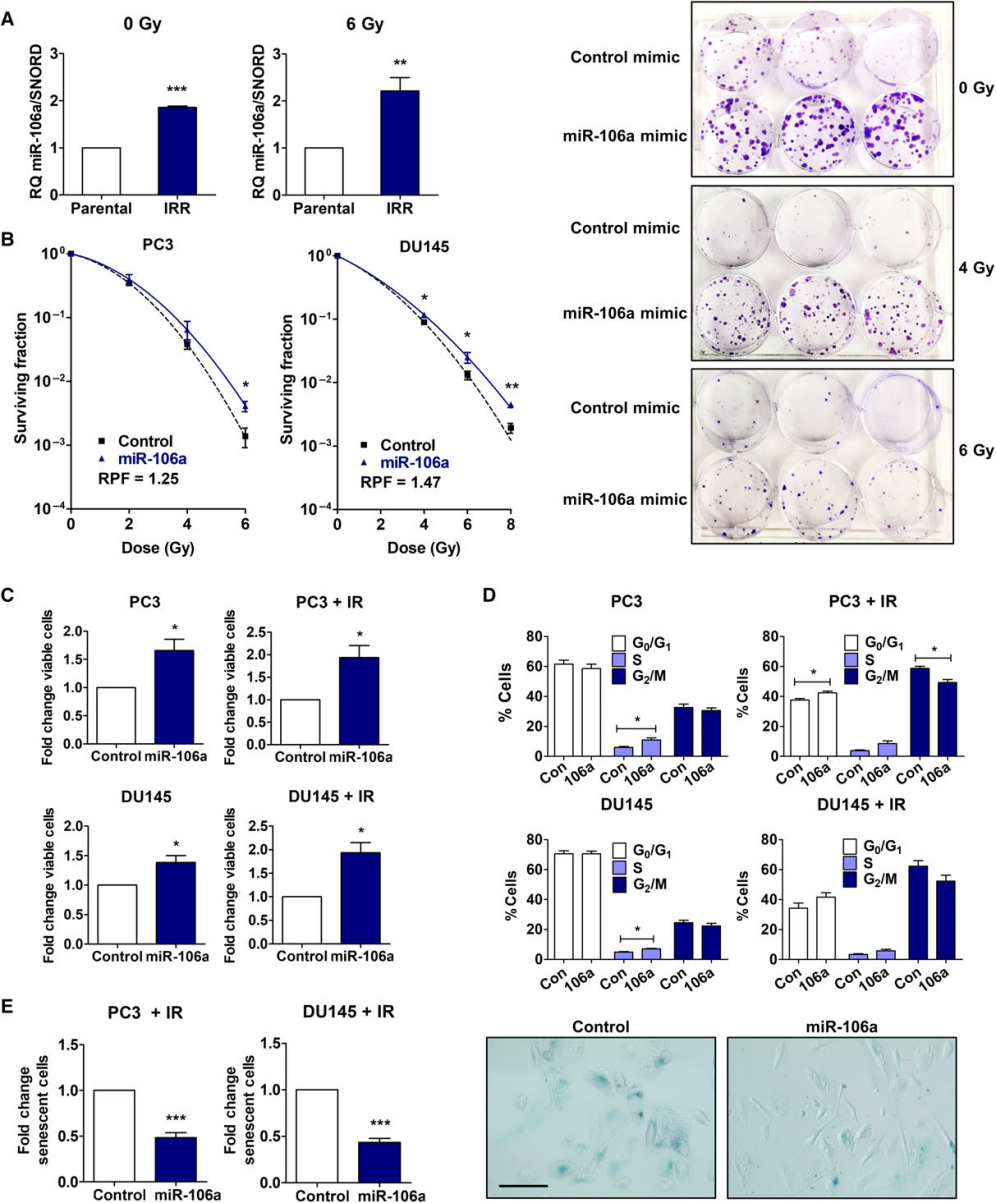
miR‐106a promotes an aggressive phenotype in prostate cancer. (A) PC3 parental and radiation‐resistant (IRR) cells treated with mock irradiation (IR) (0 Gy) or 6 Gy IR, and analyzed for miR‐106a expression by qRT‐PCR. (B) Clonogenic survival assays were performed in PC3 and DU145 cells transiently transfected with control/miR‐106a mimic to assess survival after IR. Surviving fraction was fitted to the linear quadratic equation and represented in logarithmic scale. Representative images of DU145 control/miR‐106a mimic clonogenic plates irradiated at 4 Gy and 6 Gy. (C) Proliferation of PC3 and DU145 control/miR‐106a cells at 4d after mock‐IR (0 Gy), and 5d after 8 Gy IR. (D) Cell cycle profile of PC3 and DU145 control/miR‐106a cells was assessed with mock‐IR and 24 h after IR (6 Gy for PC3, 10 Gy for DU145). (E) SA‐β‐galactosidase assay was performed with PC3 and DU145 control/miR‐106a cells 7d after 6 Gy IR. Representative images show PC3 control/miR‐106a‐irradiated cells stained for SA‐β‐galactosidase (scale bar = 100 μm). Mean, SEM and statistical significance are denoted; *P <* *0.05; **P <* *0.01; ***P <* *0.001; *n* = 3 biological replicates.

To identify miRNA involved in radioresistant prostate cancer, we measured miRNA abundance by NGS in our PC3 IRR and parental cell lines (Huang *et al*., 2013). We discovered that miR‐106a was increased 2.63‐fold in IRR cells relative to parental cells. We validated that miR‐106a is overexpressed in IRR cells with qRT‐PCR, and this was maintained after radiation treatment (Fig. 2A). Further, we observed upregulation of miR‐106a in DU145 cells following 6 Gy radiation at 30 min (Fig. S1). Chaudhry *et al*. (2013) also showed upregulation of miR‐106a expression in human lymphoblast cells after radiation. Taken together, these results suggest that miR‐106a may play an essential role in radiation response and survival.

To investigate the effect of miR‐106a on radiation survival, we performed clonogenic survival assays, the gold standard assay for radiation survival analysis. miR‐106a overexpression significantly increased surviving fraction after radiation in both PC3 (6 Gy, *P *<* *0.05) and DU145 cells (4 Gy, *P *<* *0.05; 6 Gy, *P *<* *0.05; 8 Gy, *P *<* *0.01; Fig. 2B). Clonogenic survival is represented in a logarithmic scale. We performed proliferation assays, and in support of increased clonogenic survival, both cell lines overexpressing miR‐106a showed significantly increased proliferation with mock irradiation (0 Gy) and radiation (8 Gy) (*P *<* *0.05; Fig. 2C).

miR‐106a's clonogenic survival and proliferation effects were mirrored by cell cycle distribution profiles. Flow cytometry analysis showed significantly fewer PC3 miR‐106a cells in the G_2_/M phase compared to control after irradiation (*P *<* *0.05; Fig. 2D). This suggested that miR‐106a allowed more cells to bypass the G_2_/M checkpoint and continue through the cell cycle. There were also fewer DU145 miR‐106a cells in the G_2_/M phase after radiation, although this did not achieve significance (*P *=* *0.144; Fig. 2D). In addition, a higher percentage of PC3 and DU145 cells had entered the S phase with miR‐106a overexpression compared to control (*P *<* *0.05; Fig. 2D). This trend was maintained following radiation, although this did not achieve significance (PC3, *P *=* *0.06; DU145, *P *=* *0.11; Fig. 2D). This is concordant with our proliferation data, where miR‐106a‐transfected cells showed significantly increased proliferation before and after radiation (*P *<* *0.05; Fig. 2C).

In addition to altering cell cycle distribution, miR‐106a's effect on radiation survival could be attributed to reduced cell death. The PI3K‐Akt pathway is an imperative cell survival pathway in radioresistance, and we have previously shown that miR‐95 can upregulate this pathway (Huang *et al*., 2013). However, in the context of miR‐106a, we did not observe a difference in Akt activation, indicating that upregulation of this pathway is not contributing to radioresistance in this context (Fig. S2). Considering that senescence is a predominant form of cell death after radiation in solid tumors (i.e., prostate) (Eriksson and Stigbrand, 2010; Gewirtz *et al*., 2008; Mirzayans *et al*., 2013; Wu *et al*., 2012), we evaluated miR‐106a's effect on senescence. Senescence‐associated (SA)‐β‐galactosidase assays revealed there were significantly fewer cells undergoing senescence in irradiated PC3 and DU145 miR‐106a cells compared to control (PC3, *P *<* *0.001; DU145, *P *<* *0.001; Fig. 2E). There were very few senescent cells (< 1%) in unirradiated PC3 and DU145 cells, and no difference was seen between control and miR‐106a cells (data not shown). Invasiveness, in addition to proliferation and reduced cell death, is a property of aggressive tumors. Therefore, we assayed miR‐106a's effect using the Matrigel invasion assay, but did not observe a significant change (Fig. S3).

Due to its role in radiation resistance, we evaluated miR‐106a's effects on DNA damage response (DDR). The formation and resolution of γ‐H2AX foci (phosphorylated histone‐2AX) following genotoxic stress is a well‐established approach to assay DNA double‐stranded break (DSB) repair. We therefore performed immunofluorescence microscopy to quantitate the proportion of γ‐H2AX foci between miR‐106a‐overexpressing and control cells (Methods S1). We observed increased percentage of high foci cells at 5 min following radiation and resolution of high foci by 24 hr. However, there were no significant differences between control/miR‐106a cells at any time point (Fig. S4). This suggested that altered DDR was not responsible for radiation resistance caused by miR‐106a.

### 3.3 miR‐106a increases tumor growth radioresistance *in vivo*

**Figure 3:**
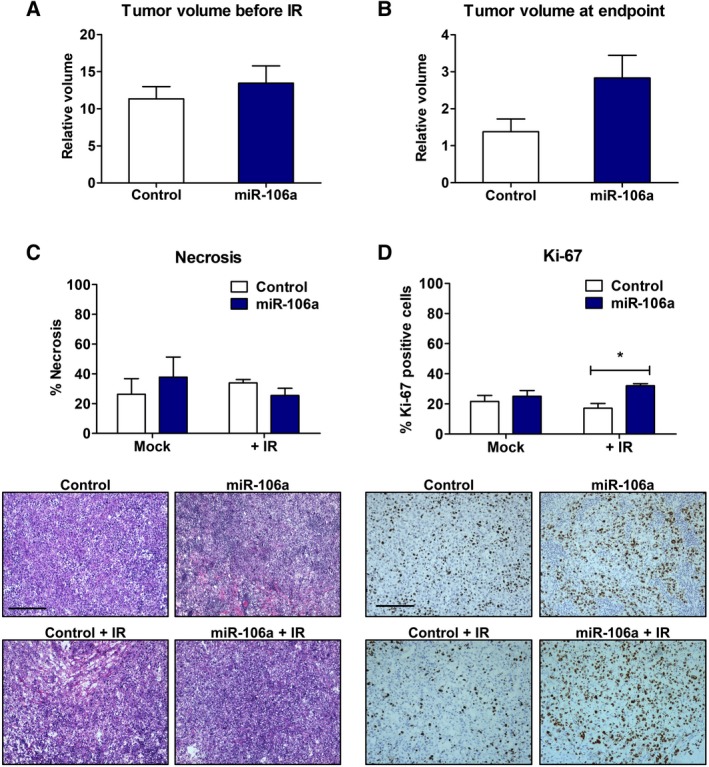
miR‐106a increases tumor growth and radioresistance *in vivo*. (A) Tumor volume of DU145 control and miR‐106a stable tumors before irradiation (IR), relative to volume at first day tumors were palpable (*n* = 14 per group). No difference was seen between control and miR‐106a stable tumors (*P *=* *0.46). (B) Endpoint (Day 37) measurement for DU145 control and miR‐106a stable tumors relative to volume at IR (Day 0) (*n* = 7 per group). A trend toward increased volume in DU145 miR‐106a‐stable compared to control tumors was seen after 6 Gy IR (*P *=* *0.06). (C) DU145‐control and DU145‐miR‐106a tumors were stained with H&E. Areas of necrosis were outlined and quantified relative to total section area. Representative images are shown below. (D) DU145‐control and DU145‐miR‐106a tumors were stained for Ki‐67. Ki‐67‐positive nuclei were quantified and plotted. Representative images are shown below (scale bar = 200 μm). Mean, SEM, and statistical significance are denoted; *P <* *0.05.

To characterize the effects of miR‐106a *in vivo*, we evaluated tumor growth in athymic nude mice. Irradiated DU145‐miR‐106a tumors displayed a trend toward larger endpoint volumes compared to control tumors (volume: control = 1.38, miR‐106a = 2.83; *P *=* *0.06; *n* = 7 per group; Fig. 3A/B). As previously discussed, 6 Gy is within the range of ionizing radiation used for SBRT treatment of prostate tumors. SBRT requires five fractions; therefore, we would expect the effect seen in our study to increase after additional radiation fractions.

To assess cellular changes, tumors were excised once they reached endpoint and processed for histological analysis. H&E staining revealed a trend toward less necrosis in DU145‐miR‐106a tumors irradiated at 6 Gy, compared to DU145‐control tumors; however, this did not achieve statistical significance (Fig. 3C). No difference in necrosis was found between unirradiated (mock) tumors. Ki‐67 staining showed that DU145‐miR‐106a tumors had significantly more proliferative cells compared to DU145‐control tumors after 6 Gy (%Ki‐67: control = 17.0989, miR‐106a = 32.0722; *P *<* *0.05; Fig. 3D). No significant difference in %Ki‐67‐positive cells was seen between unirradiated groups. Taken together, these *in vivo* results suggest that miR‐106a overexpression increases tumor growth after radiation leading to a radioresistant and aggressive phenotype.

### 3.4 miR‐106a promotes radioresistance and tumor aggression by targeting LITAF

To understand the mechanism by which miR‐106a confers radioresistance and tumor aggression, we first investigated genes previously reported as targets for miR‐106a. The critical *PTEN* tumor suppressor has been reported as a miR‐106a target. However, *PTEN* is null in the PC3 prostate cancer cell model we used, indicating that miR‐106a is not promoting radioresistance by targeting *PTEN* (Russell and Kingsley, 2003).

Retinoblastoma‐like protein 2 (RBL‐2) has been described as a target of miR‐106a in ovarian cancer (Liu *et al*., 2013). We confirmed previous findings that miR‐106a decreases RBL‐2 transcript and protein levels (Fig. S5A). However, RBL‐2 knockdown did not recapitulate increased clonogenic survival or proliferation after radiation seen with miR‐106a in PC3 or DU145 cells (Fig. S5B/C). Thus, miR‐106a was not acting through RBL‐2 to confer radioresistance, so we continued searching for miR‐106a downstream targets.

We performed a mRNA abundance microarray analysis using DU145 cells transfected with control/miR‐106a mimics. Affymetrix data were deposited to the GEO database. Of the 12 tumor suppressor gene candidates, five were previously described to be involved in proliferation, cell death, or radiation response in various cancer types: *LITAF, RASSF2, PHLPP2*,* RUNX3*, and *TP53INP1* (Huang *et al*., 2009; Jiang *et al*., 2010; Sakakura *et al*., 2007; Sandor *et al*., 2015; Shi *et al*., 2016; Wei *et al*., 2012; Yamamura *et al*., 2006; Zhou *et al*., 2011). Expression levels between PC3 and DU145 control and miR‐106a cells were compared using qRT‐PCR (Fig. S5D). RUNX3 and TP53INP1 were not consistently downregulated in both cell lines. PHLPP2 is an Akt phosphatase (Jiang *et al*., 2010); however, as miR‐106a does not promote radioresistance through upregulation of Akt signaling (Fig. S2), we did not focus on PHLPP2 further. RASSF2 is a pro‐apoptotic effector protein in the RAS/PI3K/Akt pathway, so similar to PHLPP2, we did not focus on this gene any further (Donninger *et al*., 2010).

**Figure 4:**
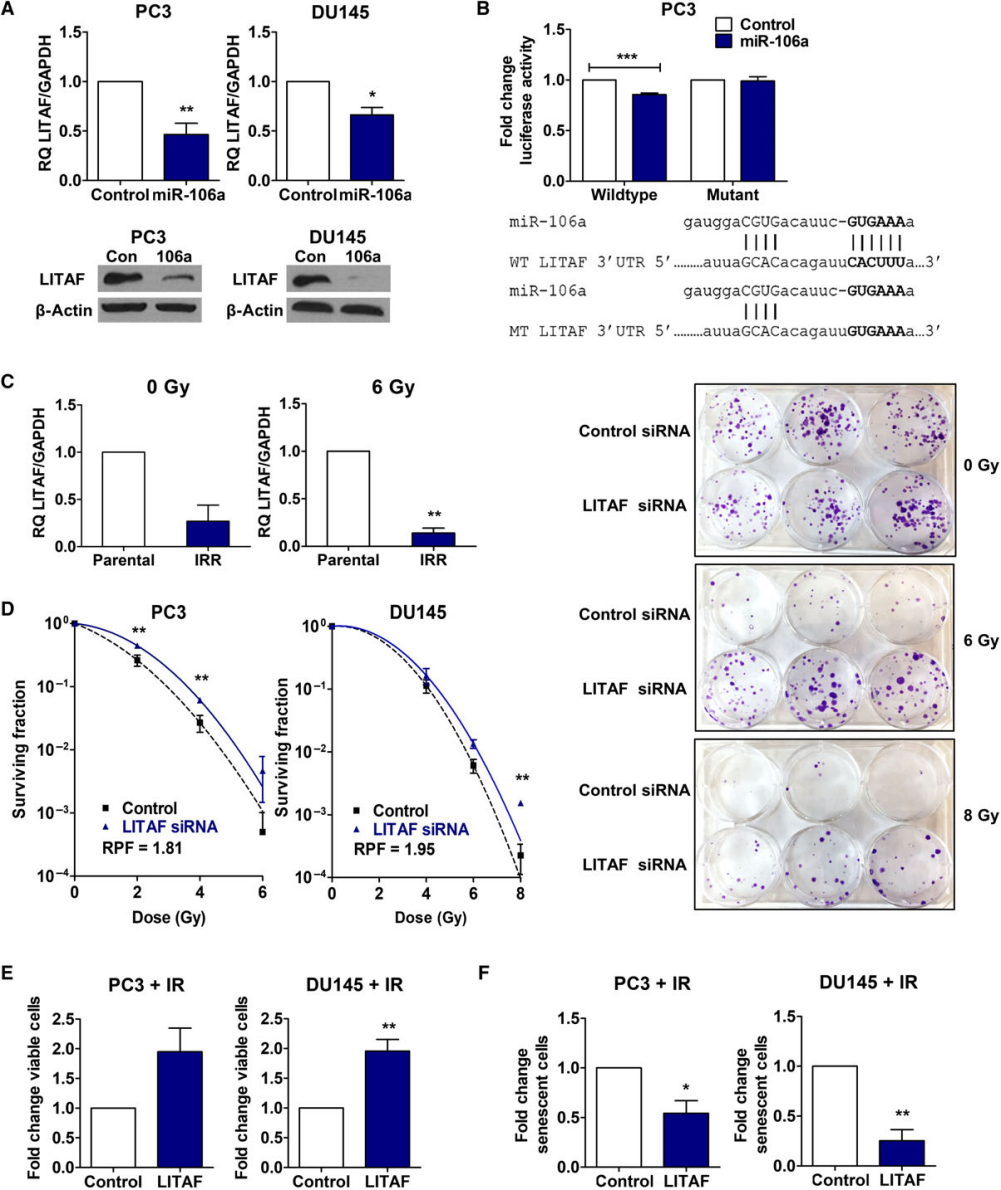
Lipopolysaccharide‐induced TNF‐α factor is a target of miR‐106a and confers a radioresistant and aggressive phenotype when inhibited. (A) qRT‐PCR of LITAF mRNA expression (normalized to GAPDH), and representative western blot of LITAF protein expression in PC3 and DU145 control/miR‐106a cells. (B) Wild‐type (WT) or mutant (MT) LITAF 3′UTR luciferase reporter assay in PC3 control/miR‐106a mimic cells with firefly luciferase activity normalized to β‐galactosidase activity. (C) PC3 parental and radiation‐resistant (IRR) cells treated with mock irradiation (IR) (0 Gy) or 6 Gy IR, and analyzed for LITAF expression by qRT‐PCR. (D) Clonogenic survival assays were performed in PC3 and DU145 cells transiently transfected with control/LITAF siRNA to assess survival after IR. Surviving fraction was fitted to the linear quadratic equation and represented in logarithmic scale. Representative image of DU145 control/LITAF siRNA clonogenic plate at 6 Gy or 8 Gy. (E) Proliferation of PC3 and DU145 control/LITAF siRNA cells 5d after 8 Gy IR. (F) SA‐β‐galactosidase assay was performed with PC3 and DU145 control/LITAF siRNA cells 7d after 6 Gy IR. Mean, SEM, and statistical significance are denoted; *P <* *0.05; **P <* *0.01; ***P <* *0.001; *n* = 3 biological replicates.

Lipopolysaccharide‐induced TNF‐α factor mRNA was significantly downregulated in PC3 and DU145 miR‐106a cells relative to control cells (PC3, RQ = 0.46, *P *<* *0.01; DU145, RQ = 0.66, *P *<* *0.05; Fig. 4A), which was confirmed at the protein level (Fig. 4A). To determine whether LITAF was a putative target of miR‐106a, we evaluated the functional interaction of miR‐106a and LITAF 3′UTR. We confirmed miR‐106a had one predicted binding site in the LITAF 3′UTR using TargetScan. PC3 cells were transiently co‐transfected with wild‐type LITAF 3′UTR luciferase reporter plasmid, and control or miR‐106a mimic. PC3 miR‐106a cells showed significantly decreased luciferase activity compared to PC3 control (*P *<* *0.001; Fig. 4B). PC3 cells co‐transfected with LITAF 3′UTR containing mutations in the predicted miR‐106a binding site showed no significant difference in luciferase activity between control and miR‐106a cells. This suggested that LITAF is a direct target of miR‐106a.

To identify whether LITAF is involved in radioresistant prostate cancer, we evaluated whether LITAF expression may also be perturbed in PC3 IRR cells compared to parental. We discovered that LITAF was decreased in PC3 IRR cells relative to parental cells with and without radiation (0 Gy, *P *=* *0.05; 6 Gy, *P *<* *0.01; Fig. 4C). LITAF expression showed the opposite trend as miR‐106a, providing further support that miR‐106a radioresistance is conferred through LITAF knockdown.

Furthermore, we characterized the consequences of LITAF knockdown with siRNA (Fig. S6) to confirm that it recapitulated the phenotype of miR‐106a overexpression. LITAF siRNA‐treated cells had a significantly higher proportion of surviving cells following radiation, compared to control PC3 cells (2 Gy, *P *<* *0.01; 4 Gy, *P *<* *0.01; Fig. 4D) and DU145 cells (8 Gy, *P *<* *0.01; Fig. 4D). These cells also showed increased proliferation after radiation compared to control cells (PC3, *P *=* *0.08; DU145, *P *<* *0.01; Fig. 4E), and a significantly lower percentage of cells undergoing senescence (PC3, *P *<* *0.05; DU145, *P *<* *0.01; Fig. 4F). Similar to miR‐106a, PC3 LITAF siRNA cells did not have any significant effect on DNA DSB repair (Fig. S4).

**Figure 5:**
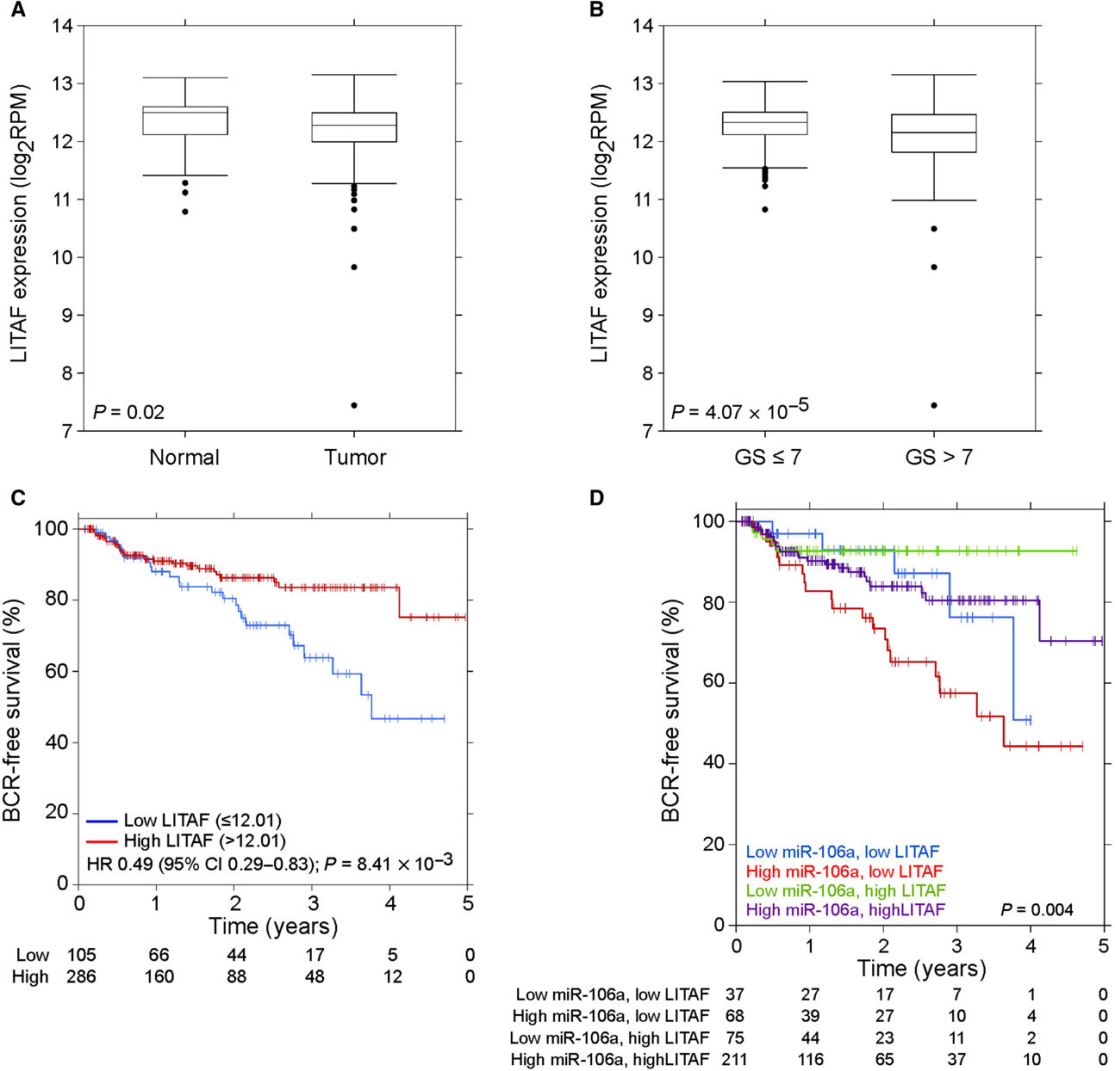
Lipopolysaccharide‐induced TNF‐α factor is aberrantly expressed in human prostate tumors. (A) LITAF shows the opposite trend as miR‐106a expression, with significantly lower expression in prostate cancer compared to normal tissue (*P *=* *0.02; *n* = 487 tumor and *n* = 52 normal matched; two‐sided Wilcoxon test). (B) LITAF shows lower expression in Gleason score (GS) >7 tumors compared to GS ≤7 tumors (*P *=* *4.07 ×  10^‐5^; *n* = 287 for GS ≤7 and *n* = 200 for GS >7; two‐sided Wilcoxon test). (C) Low LITAF expression is associated with lower BCR‐free survival at 5 years (391 tumors; *P *=* *8.41 ×  10^−3^). (D) The relationship between BCR‐free survival and four combinations of miR‐106a and LITAF expression is shown. High miR‐106a and low LITAF expression show the lowest BCR‐free survival (391 tumors; *P *=* *0.004; log‐rank test).

TCGA dataset analysis revealed that LITAF was significantly downregulated in prostate cancer compared to matched normal samples (*P *=* *0.02, two‐sided Wilcoxon test; Fig. 5A). LITAF showed significantly lower expression in clinical high‐grade (Gleason >7) compared to low‐grade (Gleason ≤7) tumors (*P *=* *4.07 × 10^−5^, two‐sided Wilcoxon test; Fig. 5B). This suggests that LITAF downregulation, likely by miR‐106a, is involved in prostate cancer tumorigenesis and tumor progression. Furthermore, using Kaplan‐Meier survival curves, we determined that low LITAF expression is associated with decreased BCR‐free survival at 5 years (391 tumors; *P *=* *8.41 × 10^−3^; Wald test; Fig. 5C). The combination of high miR‐106a and low LITAF was associated with the lowest BCR‐free survival of all four subgroups (*P *=* *0.004; log‐rank test; Fig. 5D).

Taken together, these results indicate that LITAF is a target of miR‐106a, and LITAF knockdown phenocopies radioresistance seen in miR‐106a‐overexpressing cells. This also highlights a novel role for LITAF in prostate cancer radioresistance and disease progression.

### 3.5 ATM is a downstream effector of miR‐106a and LITAF

**Figure 6:**
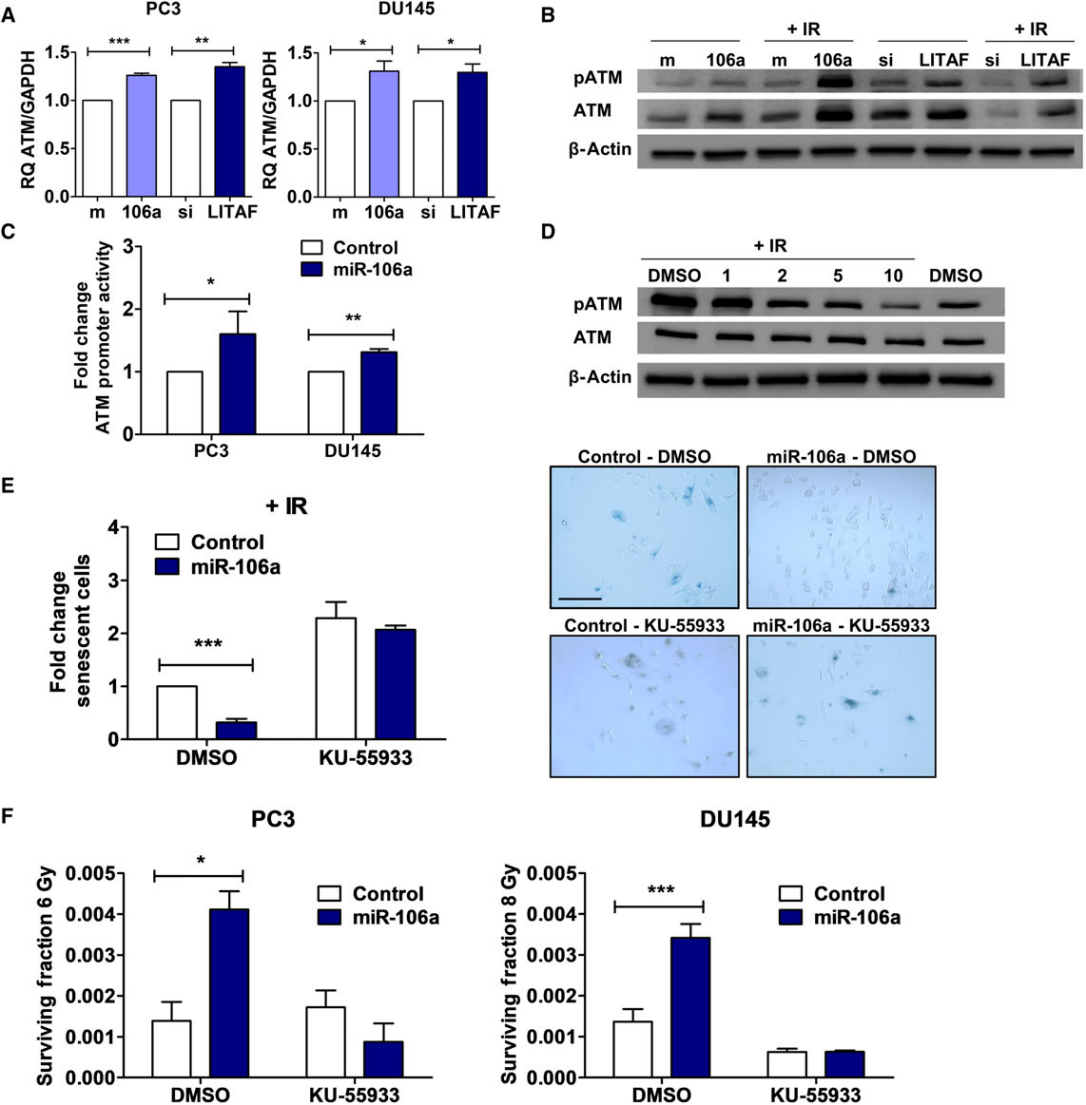
miR‐106a and LITAF regulate ATM expression to confer radioresistance. (A) qRT‐PCR for ATM mRNA expression (normalized to GAPDH) in PC3 and DU145 control(m)/miR‐106a(106a) mimic and control(si)/LITAF siRNA cells. (B) Representative western blot for phospho‐ATM (pATM), total ATM, and β‐actin (loading control) in PC3 control/miR‐106a mimic and control/LITAF siRNA cells at 0 Gy and 30 min after 6 Gy irradiation (IR). (C) ATM promoter luciferase in PC3 and DU145 control/miR‐106a mimic cells. (D) Representative western blot for pATM, total ATM, and β‐actin from PC3 cells were treated with DMSO only (vehicle), 1, 2, 5, and 10 μm of KU‐55933 2 hr before exposure to 6 Gy IR, and baseline at 0 Gy with DMSO only. (E) SA‐β‐galactosidase assay was performed with DU145 control/miR‐106a cells 7d after 6 Gy IR. Cells were treated with DMSO and 2 μm KU‐55933. Representative images show DU145 control/miR‐106a cells after 6 Gy stained for SA‐β‐galactosidase with DMSO or KU‐55933 (scale bar = 200 μm). (F) Clonogenic survival assays were performed with DU145 cells transfected with control and miR‐106a mimic. PC3 and DU145 cells were treated with DMSO or 2 μm KU‐55933 (KU) ATM inhibitor 2 h prior to 6 Gy or 8 Gy radiation, respectively. Mean, SEM, and statistical significance are denoted; *, *P *<* *0.05; **, *P *<* *0.01; ***, *P *<* *0.001; *n* = 3 biological replicates.

Due to miR‐106a and LITAF's effects on senescence and radiation survival, we evaluated senescence markers to understand their mechanism of radioresistance. The key senescence markers p53, p16, p21, and pRb are null or mutated in PC3 and DU145 cell lines (Chang *et al*., 2014; Russell and Kingsley, 2003; Sobel and Sadar, 2005). We therefore looked at ATM, another marker of senescence (Herbig *et al*., 2004). We performed qRT‐PCR and western blot to evaluate ATM expression in PC3 and DU145 cells treated with control/miR‐106a mimic, and control/LITAF siRNA. We found that both miR‐106a overexpression and LITAF knockdown in PC3 and DU145 cells resulted in significantly increased ATM transcript levels compared to control cells (PC3: miR‐106a, *P *<* *0.001; LITAF, *P *<* *0.01; DU145: miR‐106a, *P *<* *0.05; LITAF, *P *<* *0.05; Fig. 6A). This was also seen at the protein level in PC3 (Fig. 6B) and DU145 (not shown) cells before and after irradiation. Considering this, we co‐transfected PC3 and DU145 cells with an ATM promoter luciferase construct and miR‐106a mimics. We found that both cell lines overexpressing miR‐106a significantly increased ATM promoter activity compared to cells treated with control mimic (PC3, *P *<* *0.05; DU145, *P *<* *0.01; Fig. 6C). This suggests that miR‐106a and LITAF likely act to upregulate ATM expression through transcriptional activation of the ATM promoter.

Radiotherapy resistance can result from alterations in engagement of cellular DDR. ATM is the initial sensor of DNA DSBs following IR. However, as described above, we did not find miR‐106a or LITAF to have any effect on DNA DSB repair (Fig. S4). Therefore, we focused on the role of ATM in radiation resistance through senescence.

**Figure 7:**
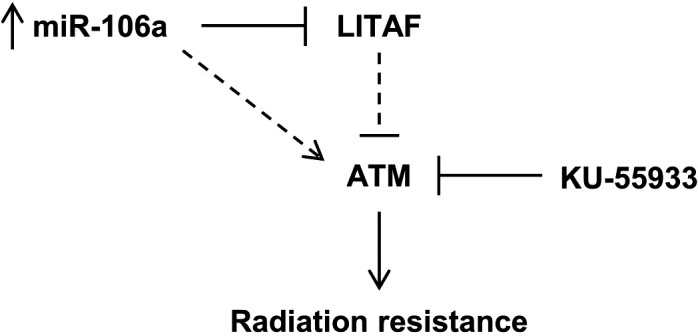
Proposed model by which miR‐106a confers radiation resistance. miR‐106a upregulates ATM expression through LITAF knockdown, to inhibit senescence and confer radioresistance in prostate cancer. KU‐55933 has been identified as a therapeutic intervention for miR‐106a‐induced radioresistance.

KU‐55933 is a highly specific ATM kinase inhibitor. We confirmed inhibition of phospho‐ATM with KU‐55933 at 2 μm with western blot after performing a dose‐response optimization study (Fig. 6D). ATM is well known for its role in senescence, so we evaluated whether KU‐55933 treatment could resensitize miR‐106a cells to radiotherapy through induction of senescence. When miR‐106a‐overexpressing cells were treated with KU‐55933, the percentage of senescent cells increased, and cellular levels of senescence were restored to similar levels as parental cells (Fig. 6E). We performed clonogenic survival assays treating miR‐106a‐overexpressing cells with KU‐55933 and found that KU‐55933 resensitizes miR‐106a cells to radiation (Fig. 6F). These results suggest that miR‐106a, acting through LITAF, upregulates ATM to increase survival after radiation and induce radioresistance (Fig. 7). miR‐106a's effect on prostate cancer radioresistance could be therapeutically targeted through inhibition of ATM kinase activity.

## 4 Discussion

Despite advances in radiotherapy, prostate cancer recurrence is still a major clinical problem, particularly in high‐grade cancer. Thus, there is an urgent need to understand the biology of radioresistant tumors. miRNA play an imperative role in regulating hundreds of pathways simultaneously within the cell (Huntzinger and Izaurralde, 2011). By understanding how dysregulated miRNA alter tumor biology, we can develop new combination therapies to sensitize resistant tumors to radiotherapy.

miR‐106a is one of seven miRNA of the oncogenic miR‐17 family. Specifically, miR‐106a has previously been shown to be upregulated in various cancer types, including gastric, ovarian, pancreatic, colorectal, nonsmall cell lung, and prostate (Ak *et al*., 2014; Kim *et al*., 2014; Volinia *et al*., 2006). Dysregulation of miR‐106a expression has been associated with chemotherapy resistance and metastasis (Ak *et al*., 2014; Kim *et al*., 2014; Li *et al*., 2014). Despite these findings, miR‐106a's role in prostate cancer radiation response is, to the best of our knowledge, unknown.

Analyzing the TCGA dataset (Cancer Genome Atlas Research Network, 2015), we found that miR‐106a is significantly overexpressed in human prostate tumors compared to normal controls, in agreement with Volinia *et al*. (2006). An advantage to our analysis with the TCGA dataset is the increased number of patient samples available. We also discovered that increased miR‐106a expression correlated with higher Gleason score, suggesting that miR‐106a is involved in prostate cancer tumorigenesis and progression. Tumors of high Gleason score are more likely to recur following treatment and are more resistant to therapies upon recurrence (Chang *et al*., 2014). Furthermore, high miR‐106a expression was found to be a predictor of decreased BCR‐free survival at 5 years after radical prostatectomy. For miR‐106a's downstream target LITAF, it was also found that low LITAF expression is a predictor of significantly decreased BCR‐free survival. Interestingly, when assessing the relationship between BCR‐free survival and the four subgroups (i.e., combinations of expressions with miR‐106a and LITAF), we found that high miR‐106a and low LITAF expression was the strongest predictor of shortest time to BCR. This suggests that miR‐106a and LITAF may serve as putative radiotherapy response biomarkers, in addition to adverse prognostic biomarkers (i.e., high 106a + low LITAF) as seen in TCGA radical prostatectomy cohort.

Consistent with these clinical findings, this study found that miR‐106a confers an aggressive and radioresistant phenotype by increasing cell survival and proliferation after radiation, both *in vitro* and *in vivo*. This suggests that miR‐106a plays a role in radiation response and radioresistance in prostate cancer. miR‐106a has been associated with radiation response in other cancer types. miR‐106a was upregulated in a lymphoblast cell line after 2 Gy radiation (Chaudhry *et al*., 2013), and in peripheral blood cells of leukemia or lymphoma patients after myeloablative fractionated radiation (Templin *et al*., 2011). Additionally, using histopathological analysis, Conde‐Muino *et al*. observed higher miR‐106a expression in nonresponders compared to responders in patients with rectal cancer receiving chemoradiotherapy (Conde‐Muino *et al*., 2015).

We identified LITAF to be a novel target of miR‐106a, and its knockdown recapitulates miR‐106a‐induced radioresistance. LITAF has previously been described as a tumor suppressor due to its ability to decrease proliferation *in vitro* and decrease tumor growth in an *in vivo* model (Zhou *et al*., 2011). However, it is best characterized for its role in inflammatory response, as reviewed in J. Zhou *et al*. (2011). LITAF is a DNA‐binding protein and is well known for its role as a transcriptional regulator of TNF‐α (Shi *et al*., 2016). ATM and TNF‐α expression have previously been correlated: It was shown that in apoptosis, TNF‐α activated caspase‐3, which cleaved and downregulated ATM (Hotti *et al*., 2000; Smith *et al*., 1999). As LITAF is a putative transcriptional activator of TNF‐α, this outlines a possible link between LITAF and ATM. We also show LITAF is inversely correlated with ATM expression, although in our model this occurs independently of TNF‐α. In addition, because apoptosis is not a predominant form of cell death after radiation in prostate cancer, it is unlikely to be the mechanism underlying LITAF's regulation of ATM described in this study.

Interestingly, miR‐106a did not alter DDR as would be expected with targeting ATM. However, we noted a significant decrease in senescence, the major form of cell death after radiotherapy, and this is abrogated with ATM inhibition. Limited knowledge of ATM's role in senescence and the unexpected results from our study suggest senescence may be tissue dependent.

ATM dysregulation is common in cancer. MiRNA are known to target DDR components, and those inhibiting DDR machinery have been shown to sensitize prostate cancer cells to radiation (Hatano *et al*., 2015). For example, miR‐421 and miR‐18a target ATM, with miR‐421‐induced ATM downregulation recapitulating radiation sensitivity similar to that seen in Ataxia telangiectasia (A‐T) patients (Hu *et al*., 2010). A‐T is an autosomal recessive childhood neurodegenerative disease that is caused by biallelic mutation of ATM, leading to absence or deficiency of ATM protein or kinase function (Taylor and Byrd, 2005). A‐T patients show an extreme sensitivity to radiation. When ATM is downregulated (i.e., by miRNA) or mutated so it is no longer functional, cells are sensitized to radiation, mimicking the response of A‐T patients (Truman *et al*., 2005). In contrast, it has been shown that upregulation of ATM leads to radioresistance in castrate‐resistant prostate cancer, allowing cells to repair radiation‐induced DNA damage (Mahajan *et al*., 2012). Angele *et al*. also found that ATM protein was overexpressed in prostate cancer tissue compared to normal prostate using immunohistochemistry (Angele *et al*., 2004). In addition, they found a trend toward increased ATM expression in high‐grade (Gleason 8–10) prostate tumors compared to low‐ and intermediate‐grade (Gleason 6&7). Furthermore, the Human Protein Atlas revealed that ATM is overexpressed in prostate cancer (Uhlen *et al*., 2015). ATM overexpression has been correlated with poor response to radiotherapy and worse overall survival in rectal cancer and advanced nasopharyngeal carcinoma patients (Ho *et al*., 2016; Ko *et al*., 2016). High ATM protein levels have been associated with radioresistance in primary glioma, chordoma, and breast cancer cells (Tribius *et al*., 2001; Yin and Glass, 2011; Zhang *et al*., 2017). Fraser *et al*. found that somatic ATM mutations are associated with poor prognosis in localized prostate cancer (Fraser *et al*., 2017). These results support our data that upregulation of ATM is a mode of radiotherapy resistance in aggressive tumors. We discovered a novel role for miR‐106a and LITAF through upregulation of ATM expression. Targeting ATM is a promising therapeutic avenue to explore for radiosensitization. With the SA‐β‐galactosidase and clonogenic survival assays, we found that KU‐55933 treatment resensitizes miR‐106a‐overexpressing cells to radiation by increasing senescence. Consistent with our results, KU‐55933 has previously been found to induce senescence and cell death in breast, lung, and colon cancer cells (Crescenzi *et al*., 2008). Despite this, we cannot formally rule out that KU‐55933 does not also affect DDR at a later time than was assayed in this study.

MiR‐106a's clinical utility as a promising biomarker is beginning to be uncovered in various cancers. MiR‐106a has been upregulated in tissue samples and biofluids. Biofluids (i.e., blood, urine, and stool) are particularly promising for clinical translation, due to their noninvasive nature. In colorectal cancer, miR‐106a was elevated in stool samples from patients with adenomas and colorectal cancer compared to healthy controls (Link *et al*., 2010). In esophageal squamous cell carcinoma, miR‐106a upregulation in plasma was shown to be a diagnostic marker as part of a six‐miRNA signature (Zhou *et al*., 2017). In a study of patients with breast cancer, Wang *et al*. found that miR‐106a was upregulated in breast tumor specimens and matched sera compared to normal controls (Wang *et al*., 2010). In prostate cancer particularly, miR‐106a has been identified as a serum biomarker as part of a miRNA signature to differentiate high‐ and low‐risk prostate cancer (Alhasan *et al*., 2016). Another study that assessed a cohort of patients from Memorial Sloan Kettering Cancer Centre showed that miR‐106a is enriched in serum of patients with African American prostate cancer compared to serum of Caucasian patients (Yates *et al*., 2017). African American men are at a significantly higher risk for developing prostate cancer and have decreased outcomes compared to Caucasian men (Shenoy *et al*., 2016). With current diagnostic tools limited to PSA and Gleason score, the use of circulating miRNA could generate new predictive and/or prognostic biomarkers for improved patient outcomes. Future studies should investigate further into miR‐106a's biomarker feasibility for prediction of tumor radiation response and high‐grade disease.

## 5 Conclusions

This study has identified a new miRNA involved in prostate cancer radiotherapy resistance. miR‐106a is significantly upregulated in human prostate tumors, with increased expression in high‐grade (Gleason 8–10) compared to low‐ and intermediate‐grade tumors (Gleason 6&7). We also show for the first time a novel role for LITAF in regulating radioresistance, where knockdown of LITAF induces increased survival and proliferation after radiation. LITAF expression shows the opposite trend as miR‐106a in the TCGA dataset, and in addition, the combination of high miR‐106a + low LITAF expression predicts for BCR at 5 years after radical prostatectomy. We propose a model whereby miR‐106a targets LITAF, resulting in ATM upregulation and radioresistance. This may be independent of ATM's role in DDR. In addition, we have identified KU‐55933 as a potential therapeutic intervention in combination with radiation, to radiosensitize miR‐106a‐induced radiation‐resistant prostate cancer. Our findings identify miR‐106a and LITAF as novel modulators of radioresistance through ATM upregulation and suggest that miR‐106a may be a promising biomarker for high‐grade disease and radioresistant prostate cancer.

## Author contributions

CH developed project directions, designed and performed the majority of the experiments, acquired and analyzed results, coordinated work of co‐authors, and wrote the manuscript. SKL originated, conceived, and supervised the project, in addition to writing the manuscript. JJ performed bioinformatics analyses of the TCGA, which was supervised by PCB. JR, XH, and ST assisted with experiments and contributed to data analysis. JY and DWA provided OPERA Phenix imaging system and helped with data acquisition. JJ, XH, and PCB edited the manuscript. All authors approved the manuscript.

## Supporting information


**Table S1.** Reagent specifications.
**Fig. S1.** MiR‐106a is a radiation response miRNA.
**Fig. S2.** MiR‐106a does not cause radioresistance through activation of Akt survival pathway.
**Fig. S3.** MiR‐106a Matrigel invasion assay.
**Fig. S4.** MiR‐106a and LITAF siRNA do not affect DNA damage recognition or repair.
**Fig. S5.** Target search for miR‐106a.
**Fig. S6.** LITAF knockdown with LITAF siRNA.
**Methods S1.** γ‐H2AX microscopy.

## References

[mol212328-bib-0001] Agarwal V , Bell GW , Nam JW and Bartel DP (2015) Predicting effective microRNA target sites in mammalian mRNAs. eLife 4, e05005. 10.7554/eLife.05005 PMC4532895 26267216

[mol212328-bib-0002] Ak S , Tunca B , Tezcan G , Cecener G , Egeli U , Yilmazlar T , Ozturk E and Yerci O (2014) MicroRNA expression patterns of tumors in early‐onset colorectal cancer patients. J Surg Res 191, 113–122. 24746948 10.1016/j.jss.2014.03.057

[mol212328-bib-0003] Alhasan AH , Scott AW , Wu JJ , Feng G , Meeks JJ , Thaxton CS and Mirkin CA (2016) Circulating microRNA signature for the diagnosis of very high‐risk prostate cancer. Proc Natl Acad Sci USA 113, 10655–10660. 27601638 10.1073/pnas.1611596113 PMC5035901

[mol212328-bib-0004] Angele S , Falconer A , Foster CS , Taniere P , Eeles RA and Hall J (2004) ATM protein overexpression in prostate tumors: possible role in telomere maintenance. Am J Clin Pathol 121, 231–236. 14983937 10.1309/JTKG-GGKU-RFX3-XMGT

[mol212328-bib-0005] Cancer Genome Atlas Research Network (2015) The molecular taxonomy of primary prostate cancer. Cell 163, 1011–1025. 26544944 10.1016/j.cell.2015.10.025 PMC4695400

[mol212328-bib-0006] Chang AJ , Autio KA , Roach M 3rd and Scher HI (2014) High‐risk prostate cancer‐classification and therapy. Nat Rev Clin Oncol 11, 308–323. 24840073 10.1038/nrclinonc.2014.68 PMC4508854

[mol212328-bib-0007] Chaudhry MA , Omaruddin RA , Brumbaugh CD , Tariq MA and Pourmand N (2013) Identification of radiation‐induced microRNA transcriptome by next‐generation massively parallel sequencing. J Rad Res 54, 808–822. 10.1093/jrr/rrt014 PMC3766286 23447695

[mol212328-bib-0008] Conde‐Muino R , Cuadros M , Zambudio N , Segura‐Jimenez I , Cano C and Palma P (2015) Predictive biomarkers to chemoradiation in locally advanced rectal cancer. Biomed Res Int 2015, 921435. 26504848 10.1155/2015/921435 PMC4609421

[mol212328-bib-0009] Crescenzi E , Palumbo G , de Boer J and Brady HJ (2008) Ataxia telangiectasia mutated and p21CIP1 modulate cell survival of drug‐induced senescent tumor cells: implications for chemotherapy. Clin Cancer Res 14, 1877–1887. 18347191 10.1158/1078-0432.CCR-07-4298

[mol212328-bib-0010] Donninger H , Hesson L , Vos M , Beebe K , Gordon L , Sidransky D , Liu JW , Schlegel T , Payne S , Hartmann A *et al* (2010) The Ras effector RASSF2 controls the PAR‐4 tumor suppressor. Mol Cell Biol 30, 2608–2620. 20368356 10.1128/MCB.00208-09 PMC2876522

[mol212328-bib-0011] Eriksson D and Stigbrand T (2010) Radiation‐induced cell death mechanisms. Tumour Biol 31, 363–372. 20490962 10.1007/s13277-010-0042-8

[mol212328-bib-0101] Fraser M , Sabelnykova VY , Yamaguchi TN , Heisler LE , Livingstone J , Huang V , Shiah YJ , Yousif F , Lin X , Masella AP *et al* (2017) Genomic hallmarks of localized, non‐indolent prostate cancer. Nature 541, 359–364. 28068672 10.1038/nature20788

[mol212328-bib-0012] Gewirtz DA , Holt SE and Elmore LW (2008) Accelerated senescence: an emerging role in tumor cell response to chemotherapy and radiation. Biochem Pharmacol 76, 947–957. 18657518 10.1016/j.bcp.2008.06.024

[mol212328-bib-0013] Hamdy FC , Donovan JL , Lane JA , Mason M , Metcalfe C , Holding P , Davis M , Peters TJ , Turner EL , Martin RM *et al* (2016) 10‐year outcomes after monitoring, surgery, or radiotherapy for localized prostate cancer. N Engl J Med 375, 1415–1424. 27626136 10.1056/NEJMoa1606220

[mol212328-bib-0014] Hatano K , Kumar B , Zhang Y , Coulter JB , Hedayati M , Mears B , Ni X , Kudrolli TA , Chowdhury WH , Rodriguez R *et al* (2015) A functional screen identifies miRNAs that inhibit DNA repair and sensitize prostate cancer cells to ionizing radiation. Nucleic Acids Res 43, 4075–4086. 25845598 10.1093/nar/gkv273 PMC4417178

[mol212328-bib-0015] He L and Hannon GJ (2004) MicroRNAs: small RNAs with a big role in gene regulation. Nat Rev Genet 5, 522–531. 15211354 10.1038/nrg1379

[mol212328-bib-0016] Herbig U , Jobling WA , Chen BP , Chen DJ and Sedivy JM (2004) Telomere shortening triggers senescence of human cells through a pathway involving ATM, p53, and p21(CIP1), but not p16(INK4a). Mol Cell 14, 501–513. 15149599 10.1016/s1097-2765(04)00256-4

[mol212328-bib-0017] Ho V , Chung L , Revoltar M , Lim SH , Tut TG , Abubakar A , Henderson CJ , Chua W , Ng W , Lee M *et al* (2016) MRE11 and ATM expression levels predict rectal cancer survival and their association with radiotherapy response. PLoS ONE 11, e0167675. 27930716 10.1371/journal.pone.0167675 PMC5145179

[mol212328-bib-0018] Hotti A , Jarvinen K , Siivola P and Holtta E (2000) Caspases and mitochondria in c‐Myc‐induced apoptosis: identification of ATM as a new target of caspases. Oncogene 19, 2354–2362. 10822387 10.1038/sj.onc.1203567

[mol212328-bib-0019] Hu H , Du L , Nagabayashi G , Seeger RC and Gatti RA (2010) ATM is down‐regulated by N‐Myc‐regulated microRNA‐421. Proc Natl Acad Sci USA 107, 1506–1511. 20080624 10.1073/pnas.0907763107 PMC2824372

[mol212328-bib-0020] Huang KH , Huang SF , Chen IH , Liao CT , Wang HM and Hsieh LL (2009) Methylation of RASSF1A, RASSF2A, and HIN‐1 is associated with poor outcome after radiotherapy, but not surgery, in oral squamous cell carcinoma. Clinical Cancer Res 15, 4174–4180. 19509163 10.1158/1078-0432.CCR-08-2929

[mol212328-bib-0021] Huang X , Taeb S , Jahangiri S , Emmenegger U , Tran E , Bruce J , Mesci A , Korpela E , Vesprini D , Wong CS *et al* (2013) miRNA‐95 mediates radioresistance in tumors by targeting the sphingolipid phosphatase SGPP1. Cancer Res 73, 6972–6986. 24145350 10.1158/0008-5472.CAN-13-1657

[mol212328-bib-0022] Huang X , Taeb S , Jahangiri S , Korpela E , Cadonic I , Yu N , Krylov SN , Fokas E , Boutros PC and Liu SK (2015) miR‐620 promotes tumor radioresistance by targeting 15‐hydroxyprostaglandin dehydrogenase (HPGD). Oncotarget 6, 22439–22451. 26068950 10.18632/oncotarget.4210 PMC4673174

[mol212328-bib-0023] Huntzinger E and Izaurralde E (2011) Gene silencing by microRNAs: contributions of translational repression and mRNA decay. Nat Rev Genet 12, 99–110. 21245828 10.1038/nrg2936

[mol212328-bib-0024] Jiang P , Rao EY , Meng N , Zhao Y , Wang JJ (2010) MicroRNA‐17‐92 significantly enhances radioresistance in human mantle cell lymphoma cells. Radiat Oncol 5, 100. 21040528 10.1186/1748-717X-5-100 PMC2984457

[mol212328-bib-0025] Kim YW , Kim EY , Jeon D , Liu JL , Kim HS , Choi JW and Ahn WS (2014) Differential microRNA expression signatures and cell type‐specific association with Taxol resistance in ovarian cancer cells. Drug Design Develop Therapy 8, 293–314. 10.2147/DDDT.S51969 PMC3938445 24591819

[mol212328-bib-0026] Ko JJ , Klimowicz AC , Jagdis A , Phan T , Laskin J , Lau HY , Siever JE , Petrillo SK , Thomson TA , Rose MS *et al* (2016) ATM, THMS, and RRM1 protein expression in nasopharyngeal carcinomas treated with curative intent. Head Neck 38(Suppl 1), E384–E391. 25640951 10.1002/hed.24004

[mol212328-bib-0027] Li P , Xu Q , Zhang D , Li X , Han L , Lei J , Duan W , Ma Q , Wu Z and Wang Z (2014) Upregulated miR‐106a plays an oncogenic role in pancreatic cancer. FEBS Lett 588, 705–712. 24444603 10.1016/j.febslet.2014.01.007

[mol212328-bib-0028] Link A , Balaguer F , Shen Y , Nagasaka T , Lozano JJ , Boland CR and Goel A (2010) Fecal MicroRNAs as novel biomarkers for colon cancer screening. Cancer Epidemiol Biomark Prevent 19, 1766–1774. 10.1158/1055-9965.EPI-10-0027 PMC2901410 20551304

[mol212328-bib-0029] Liu Z , Gersbach E , Zhang X , Xu X , Dong R , Lee P , Liu J , Kong B , Shao C and Wei JJ (2013) miR‐106a represses the Rb tumor suppressor p130 to regulate cellular proliferation and differentiation in high‐grade serous ovarian carcinoma. Mol Cancer Res 11, 1314–1325. 24045973 10.1158/1541-7786.MCR-13-0131 PMC3911890

[mol212328-bib-0030] Mahajan K , Coppola D , Rawal B , Chen YA , Lawrence HR , Engelman RW , Lawrence NJ and Mahajan NP (2012) Ack1‐mediated androgen receptor phosphorylation modulates radiation resistance in castration‐resistant prostate cancer. J Biol Chemis 287, 22112–22122. 10.1074/jbc.M112.357384 PMC3381169 22566699

[mol212328-bib-0031] Mesci A , Huang X , Taeb S , Jahangiri S , Kim Y , Fokas E , Bruce J , Leong HS and Liu SK (2017) Targeting of CCBE1 by miR‐330‐3p in human breast cancer promotes metastasis. Br J Cancer 116, 1350–1357. 28419078 10.1038/bjc.2017.105 PMC5482727

[mol212328-bib-0032] Mirzayans R , Andrais B , Scott A , Wang YW and Murray D (2013) Ionizing radiation‐induced responses in human cells with differing TP53 status. Int J Mol Sci 14, 22409–22435. 24232458 10.3390/ijms141122409 PMC3856071

[mol212328-bib-0033] Russell PJ and Kingsley EA (2003) Human prostate cancer cell lines. Methods Mol Med 81, 21–39. 12725112 10.1385/1-59259-372-0:21

[mol212328-bib-0034] Sakakura C , Miyagawa K , Fukuda KI , Nakashima S , Yoshikawa T , Kin S , Nakase Y , Ida H , Yazumi S , Yamagishi H *et al* (2007) Frequent silencing of RUNX3 in esophageal squamous cell carcinomas is associated with radioresistance and poor prognosis. Oncogene 26, 5927–5938. 17384682 10.1038/sj.onc.1210403

[mol212328-bib-0035] Sandor N , Schilling‐Toth B , Kis E , Fodor L , Mucsanyi F , Safrany G and Hegyesi H (2015) TP53inp1 gene is implicated in early radiation response in human fibroblast cells. Int J Mol Sci 16, 25450–25465. 26512655 10.3390/ijms161025450 PMC4632809

[mol212328-bib-0036] Shenoy D , Packianathan S , Chen AM and Vijayakumar S (2016) Do African‐American men need separate prostate cancer screening guidelines? BMC urology 16, 19. 27165293 10.1186/s12894-016-0137-7 PMC4862049

[mol212328-bib-0037] Shi Y , Kuai Y , Lei L , Weng Y , Berberich‐Siebelt F , Zhang X , Wang J , Zhou Y , Jiang X , Ren G *et al* (2016) The feedback loop of LITAF and BCL6 is involved in regulating apoptosis in B cell non‐Hodgkin's‐lymphoma. Oncotarget 7, 77444–77456. 27764808 10.18632/oncotarget.12680 PMC5363597

[mol212328-bib-0038] Smith GC , d'Adda di Fagagna F , Lakin ND and Jackson SP (1999) Cleavage and inactivation of ATM during apoptosis. Mol Cell Biol 19, 6076–6084. 10454555 10.1128/mcb.19.9.6076 PMC84521

[mol212328-bib-0039] Sobel RE and Sadar MD (2005) Cell lines used in prostate cancer research: a compendium of old and new lines–part 1. The Journal of urology 173, 342–359. 15643172 10.1097/01.ju.0000141580.30910.57

[mol212328-bib-0040] Taylor AM and Byrd PJ (2005) Molecular pathology of ataxia telangiectasia. J Clin Pathol 58, 1009–1015. 16189143 10.1136/jcp.2005.026062 PMC1770730

[mol212328-bib-0041] Templin T , Paul S , Amundson SA , Young EF , Barker CA , Wolden SL and Smilenov LB (2011) Radiation‐induced micro‐RNA expression changes in peripheral blood cells of radiotherapy patients. Int J Radiat Oncol Biol Phys 80, 549–557. 21420249 10.1016/j.ijrobp.2010.12.061 PMC3589812

[mol212328-bib-0042] Tribius S , Pidel A and Casper D (2001) ATM protein expression correlates with radioresistance in primary glioblastoma cells in culture. Int J Radiat Oncol Biol Phys 50, 511–523. 11380241 10.1016/s0360-3016(01)01489-4

[mol212328-bib-0043] Truman JP , Gueven N , Lavin M , Leibel S , Kolesnick R , Fuks Z and Haimovitz‐Friedman A (2005) Down‐regulation of ATM protein sensitizes human prostate cancer cells to radiation‐induced apoptosis. The Journal of biological chemistry 280, 23262–23272. 15837784 10.1074/jbc.M503701200 PMC1855286

[mol212328-bib-0044] Uhlen M , Fagerberg L , Hallstrom BM , Lindskog C , Oksvold P , Mardinoglu A , Sivertsson A , Kampf C , Sjostedt E , Asplund A *et al* (2015) Proteomics. Tissue‐based map of the human proteome. Science 347, 1260419. 25613900 10.1126/science.1260419

[mol212328-bib-0045] Volinia S , Calin GA , Liu CG , Ambs S , Cimmino A , Petrocca F , Visone R , Iorio M , Roldo C , Ferracin M *et al* (2006) A microRNA expression signature of human solid tumors defines cancer gene targets. Proc Natl Acad Sci U S A 103, 2257–2261. 16461460 10.1073/pnas.0510565103 PMC1413718

[mol212328-bib-0046] Wang F , Zheng Z , Guo J and Ding X (2010) Correlation and quantitation of microRNA aberrant expression in tissues and sera from patients with breast tumor. Gynecol Oncol 119, 586–593. 20801493 10.1016/j.ygyno.2010.07.021

[mol212328-bib-0047] Wei Q , Li YX , Liu M , Li X and Tang H (2012) MiR‐17‐5p targets TP53INP1 and regulates cell proliferation and apoptosis of cervical cancer cells. IUBMB Life 64, 697–704. 22730212 10.1002/iub.1051

[mol212328-bib-0048] Wu PC , Wang Q , Grobman L , Chu E and Wu DY (2012) Accelerated cellular senescence in solid tumor therapy. Exp Oncol 34, 298–305. 23070015

[mol212328-bib-0049] Yamamura Y , Lee WL , Inoue K , Ida H and Ito Y (2006) RUNX3 cooperates with FoxO3a to induce apoptosis in gastric cancer cells. The Journal of biological chemistry 281, 5267–5276. 16373335 10.1074/jbc.M512151200

[mol212328-bib-0050] Yates C , Long MD , Campbell MJ and Sucheston‐Campbell L (2017) miRNAs as drivers of TMPRSS2‐ERG negative prostate tumors in African American men. Frontiers in bioscience (Landmark edition) 22, 212–229. 27814612 10.2741/4482 PMC5858730

[mol212328-bib-0051] Yin H and Glass J (2011) The phenotypic radiation resistance of CD44+/CD24(‐or low) breast cancer cells is mediated through the enhanced activation of ATM signaling. PLoS ONE 6, e24080. 21935375 10.1371/journal.pone.0024080 PMC3174160

[mol212328-bib-0052] Zhang C , Wang B , Li L , Li Y , Li P and Lv G (2017) Radioresistance of chordoma cells is associated with the ATM/ATR pathway, in which RAD51 serves as an important downstream effector. Experimental and therapeutic medicine 14, 2171–2179. 28962138 10.3892/etm.2017.4736 PMC5609200

[mol212328-bib-0053] Zhou X , Wen W , Zhu J , Huang Z , Zhang L , Zhang H , Qi LW , Shan X , Wang T , Cheng W *et al* (2017) A six‐microRNA signature in plasma was identified as a potential biomarker in diagnosis of esophageal squamous cell carcinoma. Oncotarget 8, 34468–34480. 28380431 10.18632/oncotarget.16519 PMC5470983

[mol212328-bib-0054] Zhou J , Yang Z , Tsuji T , Gong J , Xie J , Chen C , Li W , Amar S and Luo Z (2011) LITAF and TNFSF15, two downstream targets of AMPK, exert inhibitory effects on tumor growth. Oncogene 30, 1892–1900. 21217782 10.1038/onc.2010.575 PMC3431012

