# Supplementary material for: miRNA‐106a and prostate cancer radioresistance: a novel role for LITAF in ATM regulation
Source: Mol Oncol. 2018 Jun 14;12(8):1324–41. doi: 10.1002/1878-0261.12328 (PMC6068351; doi:10.1002/1878-0261.12328)
Supplement: Supplementary file 1 — Table S1. Reagent specifications. Fig. S1. MiR‐106a is a radiation response miRNA. Fig. S2. MiR‐106a does not cause radioresistance through activation of Akt survival pathway. Fig. S3. MiR‐106a Matrigel invasion assay. Fig. S4. MiR‐106a and LITAF siRNA do not affect DNA damage recognition or repair. Fig. S5. Target search for miR‐106a. Fig. S6. LITAF knockdown with LITAF siRNA. Methods S1. γ‐H2AX microscopy. [file MOL2-12-1324-s001.pdf]

Supplementary Table S1. Reagent specifications. A) MiRNA mimic and siRNA sequences and specifications used in transfections. B) qRT-PCR primer sequences ordered from Invitrogen. All sequences provided in 5' – 3' orientation.

A

RNA		Sequence
Control mimic<br>QL Biosource Inc.		Sense: UUCUCCGAACGUGUCACGUTT<br>Antisense: ACGUGACACGUUCGGAGAATT
hsa-miR-106a-5p mimic<br>QL Biosource Inc.		Sense: AAAAGUGCUUACAGUGCAGGUAG<br>Antisense: ACCUGCACUGUAAGCACUUUUUU
Control siRNA (Pooled)<br>QL Biosource Inc.		Sense: UUCUCCGAACGUGUCACGUTT<br>Antisense: ACGUGACACGUUCGGAGAATT
LITAF siRNA (Pooled)<br>QL Biosource Inc.	sc-45684A:	Sense: CAUGAAUCCUCCUUCGUAU<br>Antisense: AUACGAAGGAGGAUUCAUG
	sc-45684B:	Sense: CCAUCUAACUGUGAUCAUU<br>Antisense: AAUGAUCACAGUUAGAUGG
	sc-45684C:	Sense: GGAACUCAUGUGAAUACUA<br>Antisense: UAGUAUUCACAUGAGUUCC
RBL-2 siRNA (Pooled)<br>Santa Cruz Biotech. Inc	sc-29425A:	Sense: GCAAGGUAUUGCCAAUGAATT<br>Antisense: UUCAUUGGCAAUACCUUGCTT
	sc-29425B:	Sense: GGAUCUCUGUGCCAAACUATT<br>Antisense: UAGUUUGGCACAGAGAUCCTT
	sc-29425C:	Sense: GAGACCUCAUUCAGUUCUATT<br>Antisense: UAGAACUGAAUGAGGUCUCTT

B

Primer	Sequence
SNORD61_11	Qiagen Product #218300 Catelog#MS00033705
miR-106a-5p	AAAAGUGCUUACAGUGCAGGUAG
GAPDH	F: CAGCCTCAAGATCATCAGCA; R: GTCTTCTGGGTGGCAGTGAT
LITAF	F: GGCATGAATCCTCTTCGTA; R: TGTTGCAGGAAGGACAACAC
ATM	F: GGACAGTGAGGCACAAAAT; R: GTGTCGAAGACAGCTGGTGA
RBL-2	F: ATTTGGCATGGAAACCAGAG; R: ATCTGCCCTTTCCAGGTTCT
PHLPP2	F: ATGGAGCAGACACTACCACTG; R: GCAAAGGACGAGATGTAAGTCA
RUNX3	F: TCTGTAAGGCCCAAAGTGGGTA; R: ACCTCAGCATGACAATATGTCACAA
TP53INP1	F: CCTCCAACCAAGAACCAGAA; R: GATGCCGGTAAACAGGAAAA
RASSF2	F: CTATGGCTCTGTCACCAACG; R: GCTTCTGTTTCTCACCACTCG

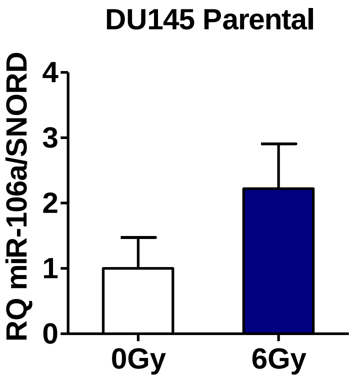

Supplementary Figure S1. MiR-106a is a radiation response miRNA. MiR-106a expression in DU145 cells treated with mock irradiation (0Gy) or 6Gy irradiation after 30 mins. Mean and SEM are denoted for three technical replicates of one biological experiment.

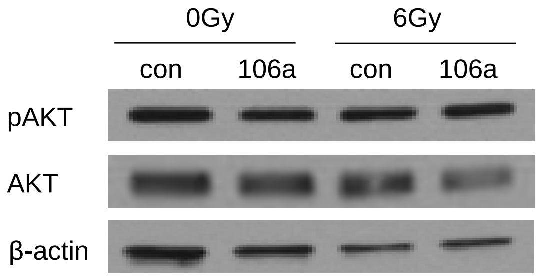

Supplementary Figure S2. **MiR-106a does not cause radioresistance through activation of Akt survival pathway.** Representative Western Blots in PC3 cell line transfected with control or miR-106a mimic.

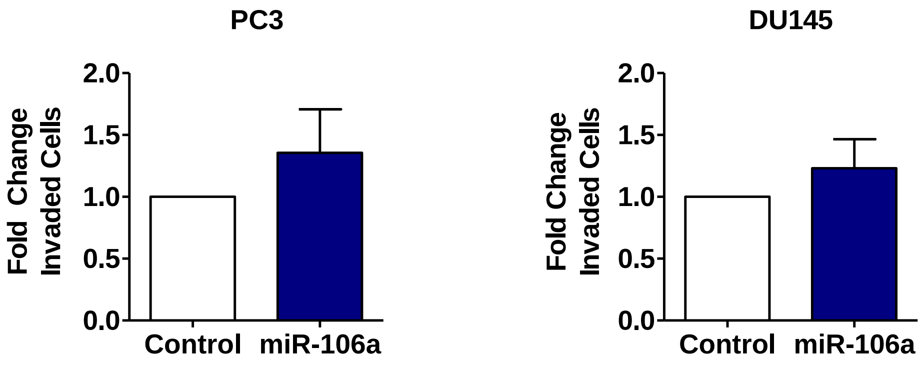

Supplementary Figure S3. MiR-106a Matrigel invasion assay. Assay was performed as previously described in Huang *et al.*, 2013. Mean, standard error, and statistical significance are denoted (PC3: P = 0.3670, DU145: P = 0.3793), n = 3 independent experiments.

A

B

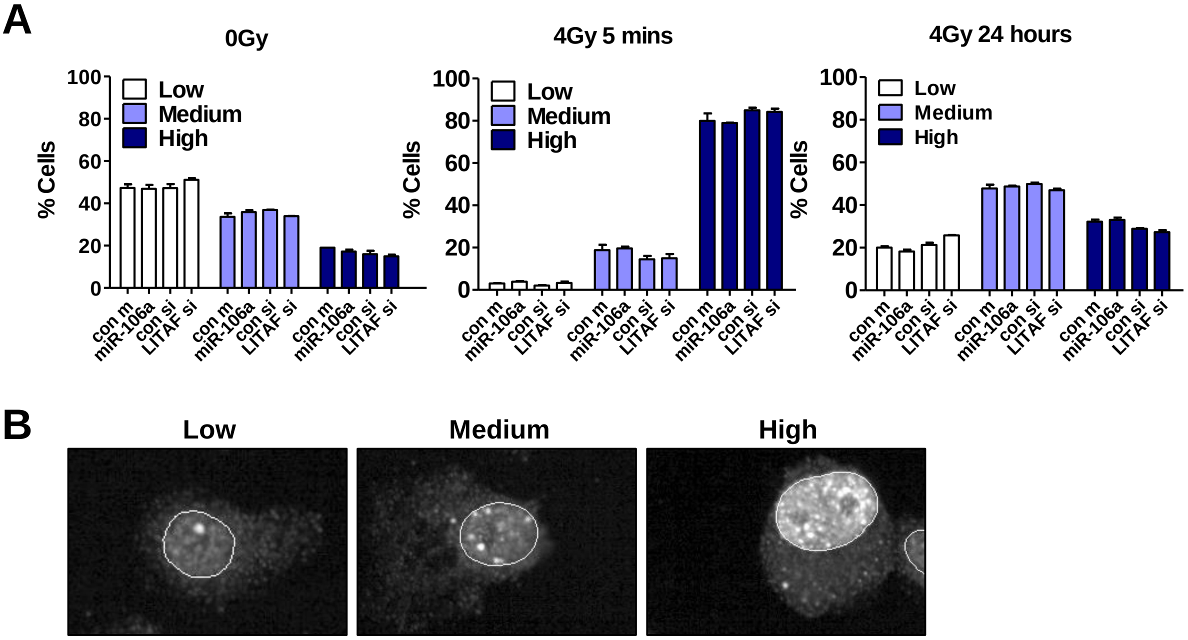

Supplementary Figure S4. MiR-106a and LITAF siRNA do not affect DNA damage recognition or repair. A) γ-H2AX foci analysis in PC3 cell line transfected with con/miR-106a mimic or con/LITAF siRNA at 0Gy, and 5mins and 24 hours after 4Gy. Mean and standard error are denoted. No statistically significant differences were seen between control and miR-106a mimic cells. B) Representative images of "Low", "Medium" and "High" γ-H2AX foci within outlined nucleus, as identified by linear classifier training in Harmony® 4.5. Experimental methods described in Supplemental Methods 1.

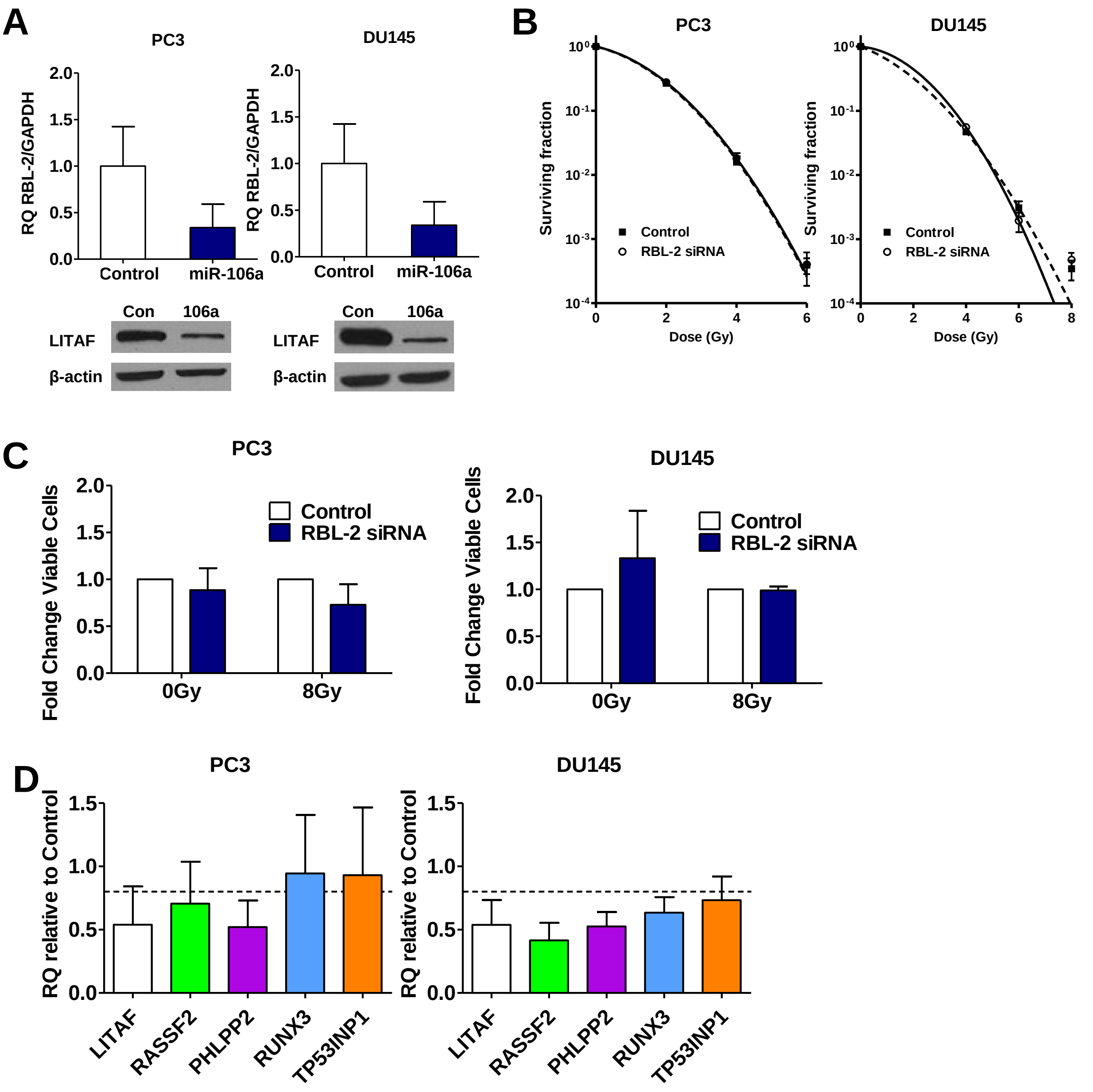

Supplementary Figure S5. Target search for miR-106a. A) qRT-PCR and representative Western blot for LITAF in PC3 and DU145 control and miR-106a cells. Mean and 95% CI are denoted. N = 3 technical replicates in one biological replicate. B) Clonogenic survival assays were performed in PC3 and DU145 cells transiently transfected with control or RBL-2 siRNA to assess survival after radiation. Surviving fraction was fitted to the linear quadratic equation. N = 3 independent experiments; 3 technical replicates within each. Mean and standard error are represented. C) Proliferation of PC3 and DU145 control or RBL-2 siRNA cells at 5 days after 8Gy radiation. N = 2 independent replicates; 3 technical replicates within each. Mean and standard error are represented. RBL-2 is not a target of miR-106a in prostate cancer radioresistance. D) MiR-106a top target qRT-PCR from microarray and *in silico* target prediction in PC3 and DU145 control and miR-106a overexpressing cells. Mean and relative quantity min/max are denoted. Dotted line representing 20% knockdown cut-off. N = 3 technical replicates in one representative biological replicate. Only LITAF, PHLPP2, and RASSF2 show >20% knockdown in both cell lines.

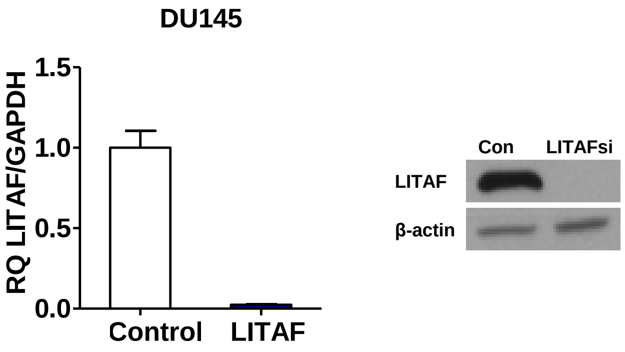

Supplementary Figure S6. LITAF knockdown with LITAF siRNA. Representative qRT-PCR and Western blot for LITAF in DU145 control/LITAF siRNA cells. Mean and 95% CI are denoted.

Supplementary Methods S1. γ-H2AX Microscopy

Mimic- and siRNA-transfected cells were seeded into Cell Carrier 96-well plates (PerkinElmer, USA) at a density of 10,000 cells per well. The following day, cells were mock-irradiated or irradiated at 4Gy and fixed 5 minutes and 24 hours after radiation. Cells were fixed with 4% paraformaldehyde in PBS for 10 mins, followed by a PBS wash before blocking in 0.1% Triton, 1% BSA, and 1% goat serum at room temperature for one hour. Mouse γ-H2AX antibody (1:1500, EMD Millipore, MilliporeSigma, Canada) was incubated overnight at 4°C. The following day, cells were washed three times with PBS, and stained with Alexa488 (1:1200, Life Technologies, Canada) goat anti-mouse antibody in 1% BSA in PBS for 1 hour at room temperature in the dark. Cells were then washed once with PBS and incubated in DAPI (1:2000) for 10 mins at room temperature in the dark. Cells were evaluated by automated high-content confocal fluorescence microscopy using Opera Phenix screening system and Harmony® 4.5 PhenoLOGIC™ analysis software (PerkinElmer, USA). Linear classifier training was performed on Harmony® 4.5. Image characteristics were assessed using γ-H2AX probe and cells were manually identified as having no/low γ-H2AX foci, “Low”; high number of γ-H2AX foci, “High”; and everything else, classified as “Medium” number of γ-H2AX foci. Features calculated by Harmony® 4.5 for linear classifier assessment include: Total Spot Area, Relative Spot Intensity, Number of Spots, Number of Spots per Area of Nucleus, SER Edge, SER Ridge, SER Hole, SER Dark, SER Saddle, SER Valley, Haralick Homogeneity 1, Haralick Sum Variance 1, Haralick Contrast 1px, and Gabor Min 2 px w2.
